# New Cancer Diagnoses Before and During the COVID-19 Pandemic

**DOI:** 10.1001/jamanetworkopen.2023.32363

**Published:** 2023-09-05

**Authors:** Kathleen M. Decker, Allison Feely, Oliver Bucher, Piotr Czaykowski, Pamela Hebbard, Julian O. Kim, Marshall Pitz, Harminder Singh, Maclean Thiessen, Pascal Lambert

**Affiliations:** 1 Paul Albrechtsen Research Institute CancerCare Manitoba, Winnipeg, Manitoba, Canada; 2 Department of Community Health Sciences, Max Rady College of Medicine, Rady Faculty of Health Sciences, University of Manitoba, Winnipeg, Manitoba, Canada; 3 Department of Epidemiology and Cancer Registry, CancerCare Manitoba, Winnipeg, Manitoba, Canada; 4 Department of Internal Medicine, Max Rady College of Medicine, Rady Faculty of Health Sciences, University of Manitoba, Winnipeg, Manitoba, Canada; 5 Department of Medical Oncology and Hematology, CancerCare Manitoba, Winnipeg, Manitoba, Canada; 6 Section of General Surgery, Department of Surgery, Max Rady College of Medicine, Rady Faculty of Health Sciences, University of Manitoba, Winnipeg, Manitoba, Canada; 7 Section of Radiation Oncology, Department of Radiology, Max Rady College of Medicine, Rady Faculty of Health Sciences, University of Manitoba, Winnipeg, Manitoba, Canada; 8 Department of Radiation Oncology, CancerCare Manitoba, Winnipeg, Manitoba, Canada

## Abstract

**Importance:**

Disruptions to health care during the COVID-19 pandemic may have led to missed cancer diagnoses. It is critical to evaluate the association between the COVID-19 pandemic and cancer incidence to address public and patient anxiety, inform recovery efforts, and identify strategies to reduce the system’s vulnerability to future disruptions.

**Objective:**

To examine the association between the COVID-19 pandemic and cancer incidence in Manitoba, Canada.

**Design, Setting, and Participants:**

A population-based cross-sectional study design was conducted using data from the Manitoba Cancer Registry and an interrupted time-series analysis. All individuals diagnosed with cancer in Manitoba, Canada, from January 1, 2015, until December 31, 2021, were included. Individuals diagnosed with breast, colon, rectal, or lung cancer were grouped by age as follows: younger than 50 years, 50 to 74 years, and 75 years and older.

**Exposures:**

COVID-19 pandemic.

**Main Outcomes and Measures:**

Age-standardized cancer incidence rates and the estimated cumulative difference between the number of cases in the absence of COVID-19 and observed (fitted) number of cancer cases.

**Results:**

A total of 48 378 individuals were included. The median (IQR) age at diagnosis was 68 (59-77) years and 23 972 participants (49.6%) were female. In April 2020, there was a 23% decrease in overall cancer incidence. Cancer incidence decreased by 46% for breast, 35% for colon, 47% for rectal, 50% for head and neck, 65% for melanoma, and 33% for endocrine cancer diagnoses and increased by 12% for hematological cancer diagnoses and 8% for diagnoses of cancers with an unknown primary site. Lung cancer incidence remained stable until December 2020 when it decreased by 11%. Brain and central nervous system and urinary cancer diagnoses decreased consistently over time from April 2020 to December 2021 by 26% and 12%, respectively. No association was observed with gynecologic (1% increase), other digestive (1% decrease), or pancreatic (7% increase) cancer incidence. As of December 2021, Manitoba had an estimated deficit of 692 (5.3%) cancers. The largest estimated deficits were for breast (273 cases, 14.1% deficit), colon (133 cases, 12.2% deficit), and lung cancers (132 cases, 7.6% deficit).

**Conclusions and Relevance:**

In this study, the COVID-19 pandemic was associated with an initial decrease in cancer diagnosis incidence followed by a recovery for most cancer sites. However, the cumulative deficit for some cancers with high fatality needs immediate attention.

## Key Points

**Question:**

Is there an association between the COVID-19 pandemic and cancer incidence in Manitoba, Canada?

**Findings:**

In this cross-sectional study including 48 378 individuals with cancer diagnoses, a significant decrease in cancer diagnosis incidence was observed in the first few months of the pandemic, particularly in breast, colon, and rectal cancer incidence. Other cancer sites showed minimal long-term changes in incidence.

**Meaning:**

The COVID-19 pandemic was associated with an initial decrease in cancer incidence followed by a return to previous incidence rates for most cancer sites.

## Teaser

This cross-sectional study evaluates associations between the COVID-19 pandemic and cancer incidence in Manitoba, Canada.

## Introduction

The COVID-19 pandemic led to the reorganization of health services to provide care for patients with COVID-19. This reorganization included greater use of virtual visits, temporary suspension of or reduction in cancer screening and diagnostic services, triaging patients with cancer for treatment based on acuity, and the redeployment of cancer care staff. Measures to address COVID-19 also varied throughout Canada and over time as COVID-19 caseloads changed.^1^

Disruptions to routine health care services may lead to missed or delayed diagnoses for individuals with suspected cancer, leading to a potential cohort of individuals with missing cancer diagnoses.^2^ Modeling studies have estimated that these individuals could experience more advanced disease at diagnosis and inferior outcomes, including decreased survival.^3,4,5^ It is therefore critical that we examine the association between the disruptions of the COVID-19 pandemic and the cancer care system in order to address public and patient anxiety, direct COVID-19 recovery efforts, and identify strategies for reducing the system’s vulnerability to future disruptions. We previously found a 23% decrease in the number of new cancer diagnoses, an 83% decrease in Papanicolaou tests, an 81% decrease in fecal occult blood tests, and a 54% decrease in the number of screening mammograms at the start of the pandemic in Manitoba.^6,7^ In this study, using a population-based cross-sectional study design and an interrupted time-series analysis, we examined the association between the COVID-19 pandemic and age-standardized cancer diagnosis incidence rates by disease site and estimated the difference between the observed and estimated number of cancer cases that may have been diagnosed in the absence of COVID-19.

## Methods

The study was conducted according to the guidelines of the Declaration of Helsinki and approved by the University of Manitoba’s Health Research Ethics Board, Manitoba Health’s Health Information Privacy Committee, and CancerCare Manitoba’s Research and Resource Impact Committee. Because data were deidentified, informed consent was not required. This study followed the Strengthening the Reporting of Observational Studies in Epidemiology (STROBE) reporting guideline.

### Setting

Manitoba, located in central Canada, has a population of approximately 1.39 million. Two-thirds of the population live in the capital city of Winnipeg. CancerCare Manitoba is the provincial cancer agency responsible for providing clinical services to all residents of Manitoba who are diagnosed with cancer. Prior to COVID-19, approximately 6000 individuals were diagnosed with cancer and 5000 received treatment or follow-up care at CancerCare Manitoba each year. The first COVID-19 case in Manitoba was identified on March 12, 2020. By the end of March, the government had implemented restrictions to mitigate the impact of virus. The peak of the first wave occurred in March 2020, the second in November 2020, and the third in May 2021.^8^ By October 1, 2021, approximately 70% of Manitoba residents were fully vaccinated.^8^

### Design and Population

We used a population-based cross-sectional study design to examine the rate of new cancer diagnoses over time before (January 2015 until February 2020) and after the start of the COVID-19 pandemic and the interventions that were implemented to mitigate its impact (April 1, 2020, to December 31, 2021). All individuals diagnoses with cancer in Manitoba from 2015 to 2021 were included.

### Data Sources

Cancer cases were determined using the Manitoba Cancer Registry, a high-quality, population-based registry that is legally mandated to collect and maintain accurate, comprehensive information about cancer diagnoses in Manitoba.^9^ The Manitoba Cancer Registry uses disease site groupings according to the *International Classification of Diseases for Oncology, Third Edition* (*ICD-0-3*). Population data were based on estimated Manitoba Health coverage provided by Manitoba Health.

### Outcomes

Outcomes included the age-standardized cancer incidence rate per 100 000, the estimated cumulative difference in the number of new cancer diagnoses, and the estimated percentage cumulative difference in the number of new cancer diagnoses. The estimated cumulative difference in new cancer diagnoses was defined as the difference between the monthly cumulative counterfactual count (the estimated number of diagnoses in the absence of COVID-19) and the monthly cumulative fitted count (ie, the observed number of diagnoses smoothed). The estimated percentage cumulative difference in the number of new cancer diagnoses was defined as the cumulative difference in the fitted count divided by the cumulative difference in the counterfactual count.

Consistent with routine reporting, cancers were categorized into the following sites: all cancers, breast, lung, prostate, colon, rectal, hematologic, urinary, unknown primary site, head and neck, brain and central nervous system, gynecologic, other digestive, melanoma, pancreatic, endocrine, and other. eAppendix 1 in Supplement 1 lists the cancers in each category and *ICD-O-3* codes. Because of the impact of the pandemic on breast and colorectal cancer screening found in prior analyses,^7^ we examined breast, colon, and rectal cancers separately for individuals younger than 50 years, 50 to 74 years (ie, eligible for screening), and 75 years and older. Rates were age standardized to the 2011 Canadian population using the direct method.

### Statistical Analysis

We used an interrupted time-series analysis that, unlike pre-post study designs, takes into consideration baseline or seasonal trends.^10^ If baseline trends are not considered, the difference between the number of diagnoses observed and the number expected in the absence of COVID-19 will be either overestimated (if there was a downward trend in diagnoses prior to the pandemic) or underestimated (if there was an upward trend in diagnoses prior to the pandemic). We compared post–COVID-19 (April 2020 onward) cancer incidence rates to counterfactual rates as if the pandemic had not occurred based on pre–COVID-19 trends (baseline rates) and quantified monthly new cancer diagnoses by cancer type.

Generalized linear models were run and selected for each cancer site based on the distribution of the data (ie, Poisson, generalized Poisson, negative binomial, gamma, or inverse gaussian). To do this, we plotted the projected mean and variance of each model and the observed mean and variance in the data.^11^ Scaled quantile residual plots were also used to evaluate the uniformity of residuals and dispersion.^12^ Each model included a binary intervention term equal to 0 during the pre–COVID-19 period and 1 during the COVID-19 period and a time term defined as the number of months since the start of the study period. Fitted values (ie, smoothed observed values) were generated using the observed values in the data frame. Counterfactual values were generated by projecting values with the binary indicator of the pandemic as 0 rather than 1. Nonlinear time and seasonality effects were accounted for in the regression model using a flexible spline function.^13^

After the generalized linear model was selected, models were fine-tuned by comparing the adjusted McFadden *R*^2^ between subsequent models.^14^ Plots were produced using observed, counterfactual, and fitted values. If the counterfactual did not follow the baseline trend, the model was modified (ie, the number of degrees of freedom for splines was reduced) until the counterfactual was consistent with the baseline trend. COVID-19–by–time interactions were considered if the plotted-fitted values in the COVID-19 period did not fit the observed data well (eg, observed values were below fitted values during the early COVID-19 period but higher during the later COVID-19 period). Time interactions were found for some cancer sites but still demonstrated poor fit (ie, fitted values did not fit observed values well). Therefore, we created multiple COVID-19 dummy variables representing different periods during the pandemic to provide a slope for each period, which enabled more accurate projections. The following R packages were used: haven, splines, Hmisc, lattice, MASS, ggplot2, car, DHARMa, multcomp, lmtest, glmmTMB, and forest plot. March 2020 was excluded from the analyses because COVID-19 restrictions and changes to visit and treatment protocols were implemented incrementally throughout March.^6,15,16^

Ratios between counterfactual and fitted estimates and 95% CIs derived from contrast statements were calculated. The estimated cumulative difference in the number of diagnoses during the pandemic was calculated as of December 31, 2021, and plotted using a forest plot. The 95% CIs for the cumulative difference estimates were calculated with parametric bootstrapping and 1000 replications. Data analyses were performed in SAS version 9.4 (SAS Institute) and R version 4.1.30.5 (R Foundation). eAppendix 2 in Supplement 1 provides R code and simulated data for conducting the interrupted time-series analysis.

## Results

**Figure 1.**
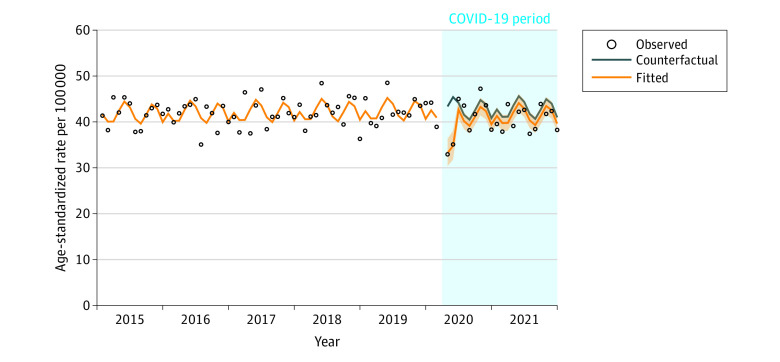
Age-Standardized Incidence Rate for All Cancers by Month From April 2020 to December 2021, Manitoba, Canada

From 2015 to 2021, there were 48 378 cases of cancer diagnosed in Manitoba (eTable 1 in Supplement 1). The median (IQR) age at diagnosis was 68 (59-77) years and 23 972 participants (49.6%) were female. Ratios between fitted and counterfactual incidence rates by month and 95% CIs are provided in eTables 2-4 in Supplement 1. Figure 1 shows observed, counterfactual, and fitted age-standardized cancer incidence rates for all cancer sites combined from January 1, 2015, to December 31, 2021 (excluding March 2020). The 95% CI of the ratio of the fitted to counterfactual value is also included. In April 2020, there was a 23% decrease in cancer incidence. By June 2020, there was no significant difference between the fitted and counterfactual cancer incidence rates.

**Figure 2.**
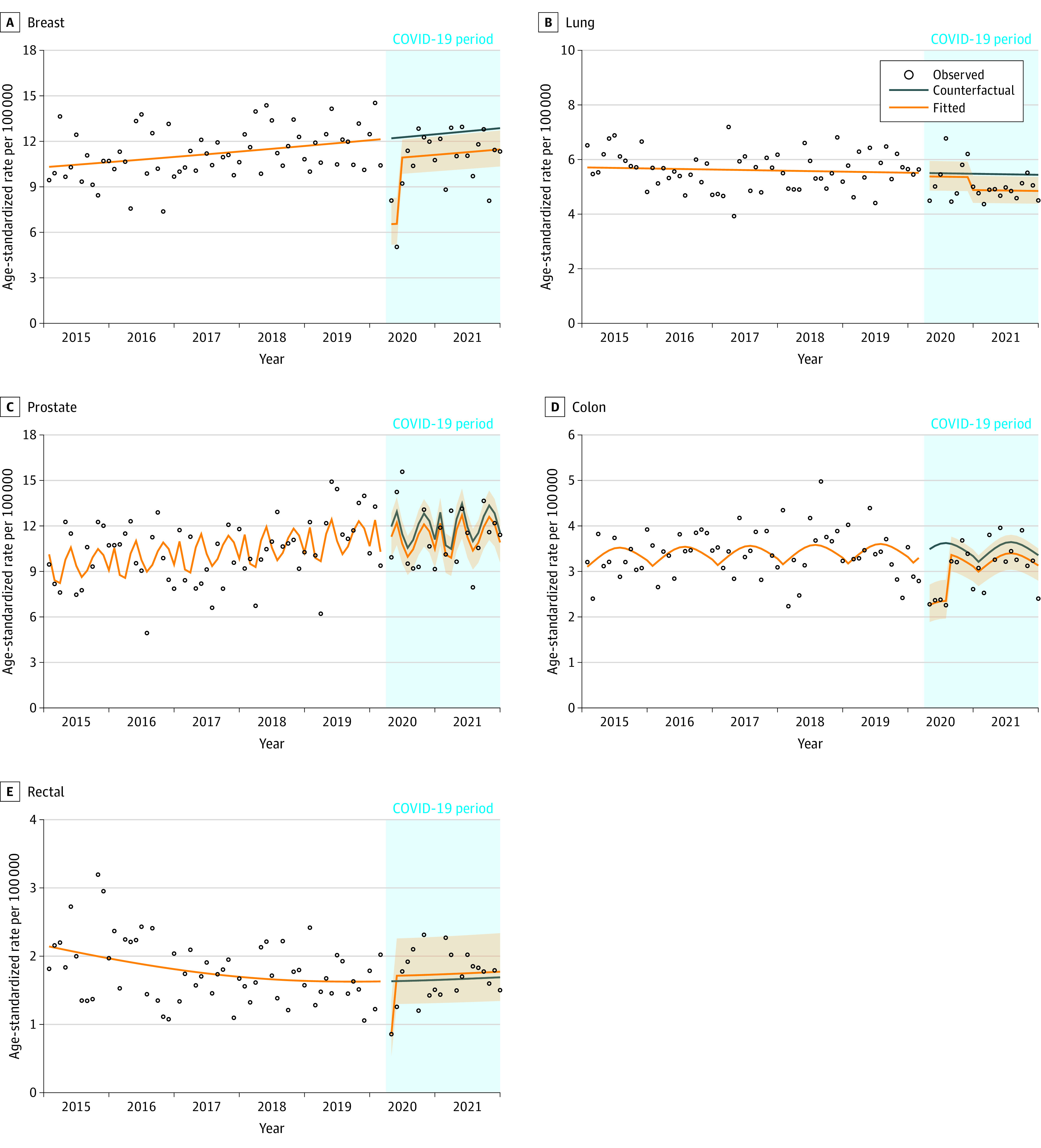
Age-Standardized Incidence Rates for Breast, Lung, Prostate, Colon, and Rectal Cancers by Month From April 2020 to December 2021, Manitoba, Canada

Figure 2 shows the observed, counterfactual, and fitted age-standardized incidence rates for breast (n = 6548), lung (n = 6477), prostate (n = 5849), colon (n = 3876), and rectal (n = 2021) cancers. in April 2020, there was a 46% decrease in breast cancer incidence. Breast cancer incidence remained 11% lower than the counterfactual until December 2021. There was no association observed with breast cancer incidence among women younger than 50 years, a decrease of 46% in April 2020 and 73% in May 2020 in women aged 50 to 74 years (screening eligible) followed by a sustained decrease below the counterfactual, and a 20% sustained decrease among women 75 years and older (eFigure 1 in Supplement 1). Lung cancer incidence remained stable until December 2020 when it decreased by 11%. Because of this decrease, we ran a post hoc subgroup analysis by age to see if the association was consistent across age groups. An association was seen only in the 75 years and older age group (eFigure 2 in Supplement 1), where lung cancer incidence decreased by 46% in April 2020. There was no association observed with prostate cancer incidence. Colon cancer incidence decreased by 35% in April 2020. By August 2020, there was no difference between the counterfactual and fitted colon cancer incidence rate. There was no association observed for colon cancer incidence among individuals younger than 50 years and a 34% decrease in those aged 50 to 74 years and those 75 years and older in April 2020 (eFigure 3 in Supplement 1). Rectal cancer incidence decreased in April 2020 by 47%. By May 2020, incidence was 5% higher than the counterfactual. Except for April 2020 for the 50 to 74 years age group for whom incidence decreased by 45%, rectal cancer incidence rate was nonsignificantly higher than the counterfactual in all 3 age groups (eFigure 4 in Supplement 1).

**Figure 3.**
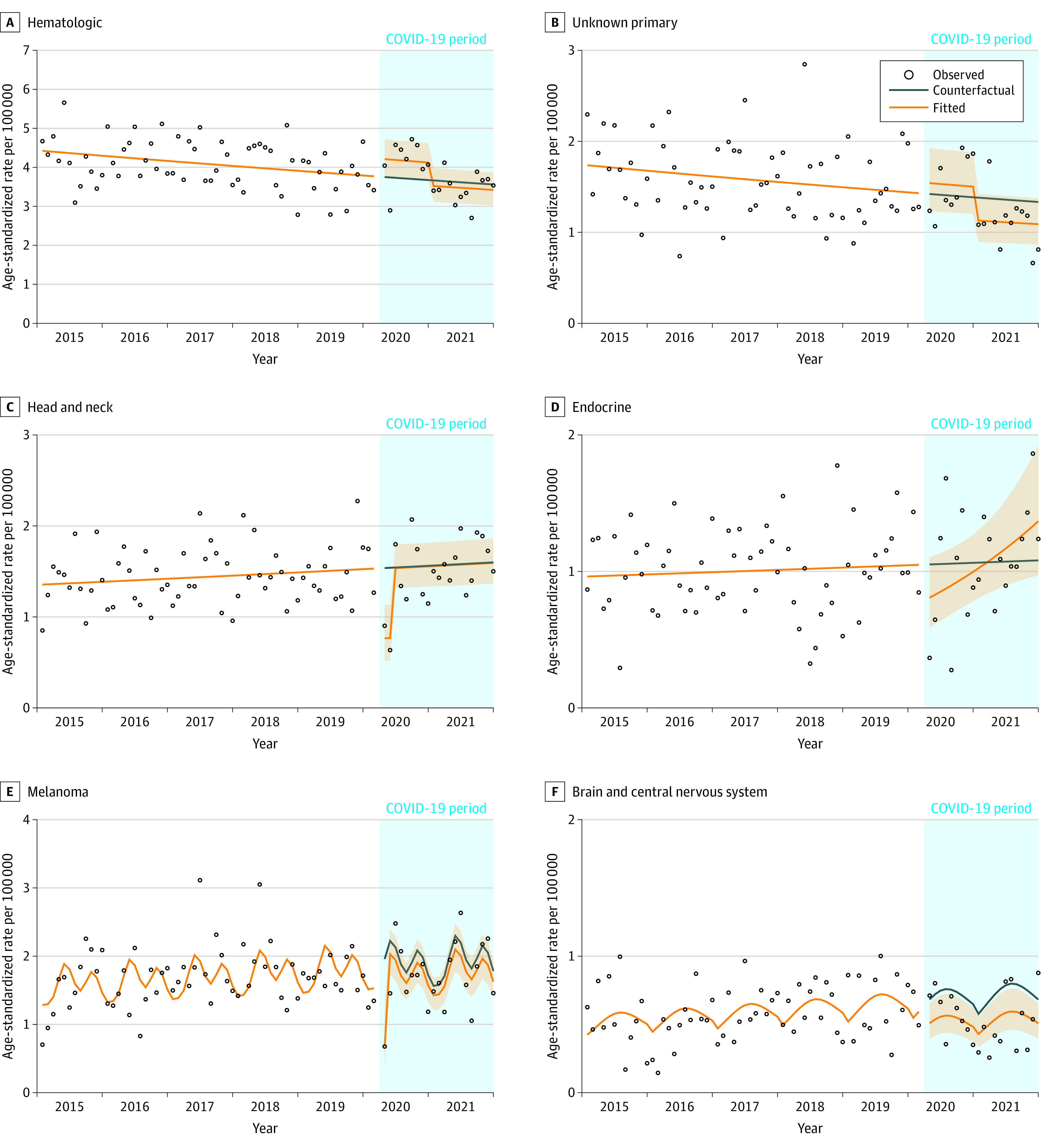
Age-Standardized Incidence Rates for Hematologic, Unknown Primary Site, Head and Neck, Endocrine, Melanoma, and Brain and Central Nervous System Cancers by Month From April 2020 to December 2021, Manitoba, Canada

**Figure 4.**
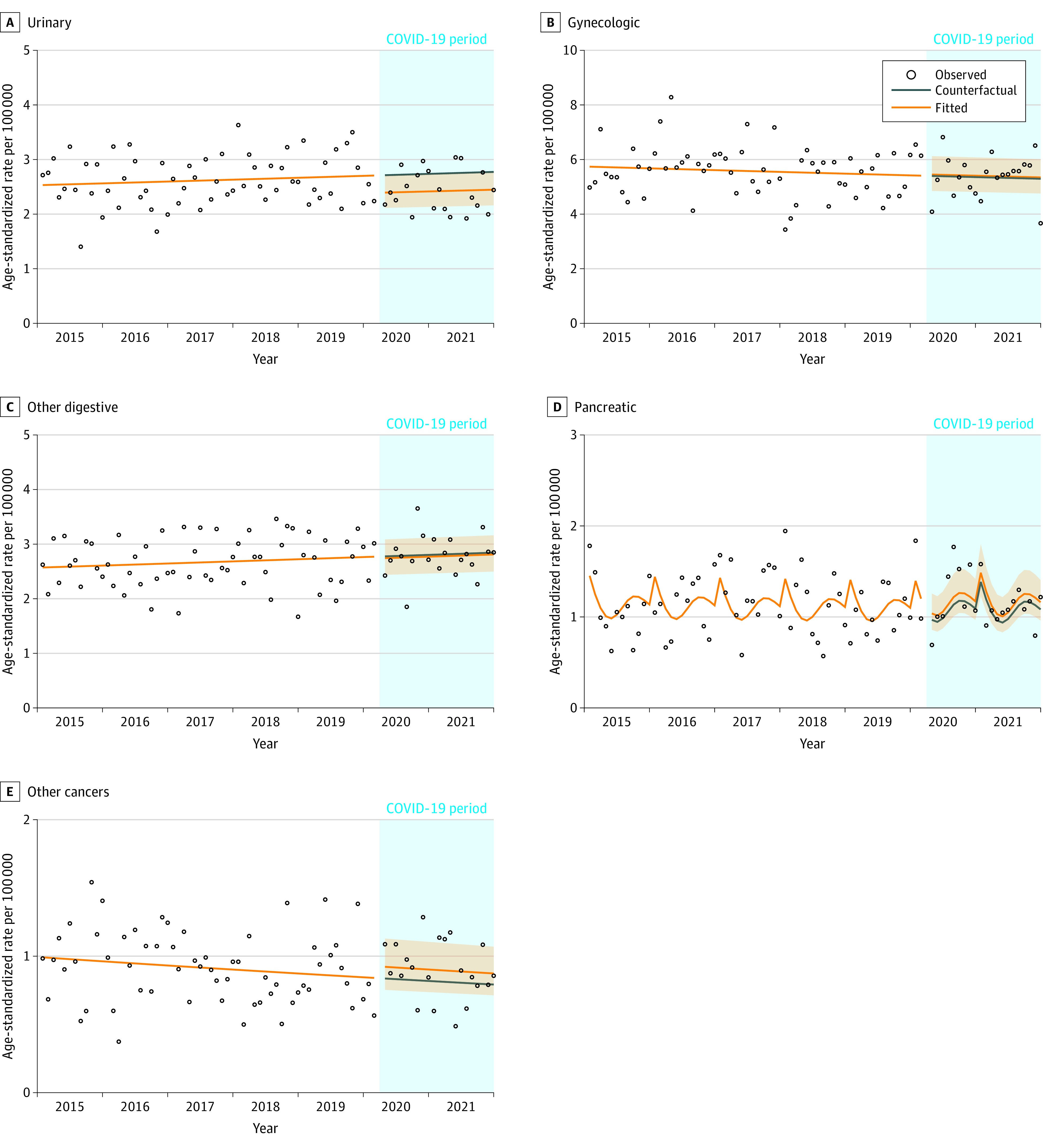
Age-Standardized Incidence Rates for Urinary, Gynecologic, Other Digestive, Pancreatic, and Other Cancers by Month From April 2020 to December 2021, Manitoba, Canada

Figure 3 shows the age-standardized observed, counterfactual, and fitted incidence rates for hematologic (n = 4655), unknown primary (n = 1764), head and neck (n = 1674), endocrine (n = 1130), melanoma (n = 1952), and brain and central nervous system (n = 662) cancers. There was a 12% increase in hematologic cancer incidence in April 2020 followed by a 4% decrease in January 2021. There was an 8% increase in the incidence of unknown primary site cancers in April 2020 followed by an 18% decrease in January 2021. Head and neck cancer incidence decreased by 50% in April 2020, but this increase was no longer significant by June 2020. Endocrine cancer incidence decreased by 33% in April 2020 and then slowly increased over time to 27% higher than the counterfactual in December 2021. Melanoma decreased by 65% in April 2020, then increased to 9% below the counterfactual in May 2020. From April 2020 to December 2021, brain and central nervous system incidence decreased by 26%. Urinary cancer incidence (n = 2982) decreased by 12% (Figure 4). There was no association observed with gynecologic (n = 3228), other digestive (n = 3164), pancreatic (n = 1352), or cancers combined into the other category (n = 1053) (Figure 4).

**Figure 5.**
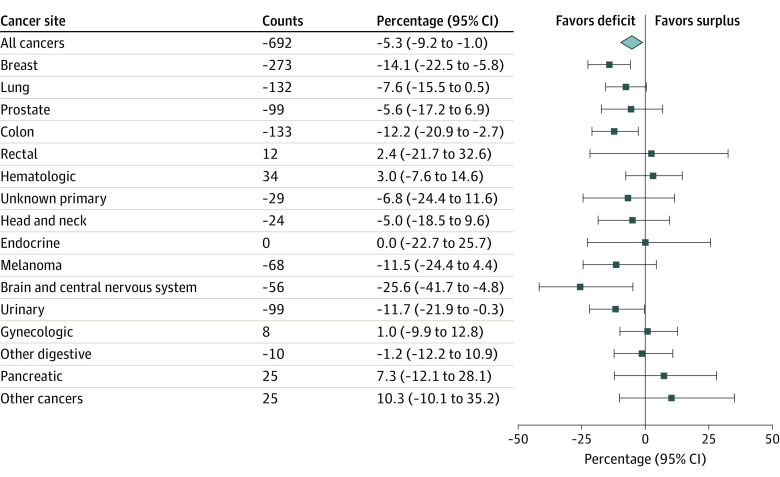
Estimated Cumulative Difference and Percentage Cumulative Difference Between the Fitted and Counterfactual Number of Cancer Cases by Cancer Site as of December 2021, Manitoba, Canada

Figure 5 and eTable 5 in Supplement 1 show the estimated cumulative difference and percentage cumulative difference between the fitted and counterfactual number of cancer diagnoses. As of December 2021, Manitoba had 692 (5.3%) fewer cancers diagnosed than expected in the absence of COVID-19. The largest estimated differences were for breast (273 cases, 14.1% deficit), colon (133 cases, 12.2% deficit), lung (132 cases, 7.6% deficit), prostate (99 cases, 5.6% deficit), urinary (99 cases, 11.7% deficit), melanoma (68 cases, 11.5% deficit), and brain and central nervous system (56 cases, 25.6% deficit) cancers. There was an estimated surplus in the number of cancer cases for rectal (12 cases, 2.4% surplus), hematologic (34 cases, 3.0% surplus), gynecologic (8 cases, 1.0% surplus), pancreatic (25 cases, 7.3% surplus), and cancers combined into the other category (25 cases, 10.3% surplus). There was no deficit or surplus for endocrine cancers.

## Discussion

In this cross-sectional study, we observed a decrease in overall age-standardized cancer diagnosis incidence and incidence for most cancer sites in Manitoba at the beginning of the COVID-19 pandemic. Longer-term decreases in incidence and cumulative deficits in the number of diagnoses were seen for breast, lung, prostate, colon, urinary, melanoma, and brain and central nervous system cancers. The association between the COVID-19 pandemic early on and breast and colon cancer incidence in individuals aged 50 to 74 years and the cumulative deficits may be explained by reductions to Manitoba’s breast and colorectal cancer screening programs during April and May 2020^7^ and reductions in the rate of colonoscopies in the province. The increase in rectal cancer incidence above the counterfactual in May 2020 is likely related to the use of a central endoscopy waitlist for most endoscopies performed in Winnipeg and the prompt and appropriate triaging of individuals with suspected rectal cancer. The decrease in breast cancer incidence among women 75 years and older may be related to reductions in diagnostic mammography availability, reluctance to seek medical care during the pandemic, or the impact of COVID-19 on mortality among older adults.^17,18^

The association between the COVID-19 pandemic and lung cancer incidence differed from breast and colorectal cancers. Lung cancer incidence dropped in December 2020 (wave 2) but only among individuals 75 years and older. This may be partly due to the increased rate of COVID-19 infections in Manitoba in the second and third waves leading to a higher number of deaths among individuals with undiagnosed lung cancer. These individuals were more vulnerable in general as well as to severe COVID-19 outcomes because of advanced age and comorbidities.^19,20^ These individuals may also have been reluctant to seek health care.^21^

Head and neck and melanoma cancer incidence also decreased at the start of the pandemic, likely due to reductions in primary care visits, but returned to prepandemic levels quickly. The decrease in urinary cancer incidence persisted over time without any observed recovery. This may reflect reduced access to cystoscopy, challenges diagnosing and managing urothelial tumors throughout the COVID-19 pandemic, or a decrease in the incidental diagnosis of kidney cancers because of reduced abdominal imaging availability. Brain and central nervous system and endocrine cancer incidence also showed large decreases in incidence but because of the small number of cases, the results must be interpreted with caution. Because the pre–COVID-19 baseline for hematologic cancers is inconsistent and there was no cumulative difference by December 2021, the post–COVID-19 change in hematologic cancer incidence may be due to a random variation.

We found no association between the COVID-19 pandemic and prostate, gynecologic, other digestive, pancreatic, or other cancer incidence. Organized prostate cancer screening for asymptomatic individuals using the prostate-specific antigen test is not recommended in Canada.^22^ Because prostate-specific antigen testing is not standardized, the actual practice is variable, which leads to unstable incidence of prostate cancer diagnosis and varying rates of overdiagnosis. Hence, prostate cancer has unreliable counterfactual values. The other cancers for which no association in incidence was observed are frequently diagnosed symptomatically and often have a high fatality rate. Therefore, despite pandemic restrictions, the health care system was still able to diagnose and prioritize these cancers resulting in minimal or no cumulative differences in the number of diagnoses.

Prior studies have evaluated the association between the COVID-19 pandemic and cancer incidence in Canada^23,24,25,26,27,28^ and internationally^29,30,31^ and also found significant declines in cancer incidence early in the pandemic, although the association for each cancer site differed by jurisdiction. However, most of these studies have important limitations, including using a pre-post study design that inadequately controls for time trends and can bias the estimated differences in the number of cancer cases^23,24,25,26,27,28,29,30^; only including cancers diagnosed at a single center, resulting in a small number of cases and limited generalizability^24,25,27,30^; and having a short follow-up time that provides no information about the association between the pandemic and cancer incidence over the long term.^23,24,25,29,31^

This study includes several important strengths. First, we used population-based, high-quality cancer registry data. Second, our interrupted time-series analysis had a long preintervention period that permitted the evaluation of outcomes before the start of the COVID-19 pandemic and the inclusion of seasonality and interactions between COVID-19 pandemic onset and time in the analysis.^10^

### Limitations

This study has limitations. Although we included the first 3 waves (21 months) of the COVID-19 pandemic, the association between COVID-19 and subsequent waves as well as by area of residence and sex must still be measured. This work is ongoing. We did not adjust for multiple comparisons; hence our results are exploratory. Because the association between the COVID-19 pandemic and cancer incidence may differ between jurisdictions, our results must be interpreted within the Manitoba context. Population-based studies should be performed elsewhere that consider pre–COVID-19 trends in cancer incidence and include sufficient follow-up time. The R code and simulated data we have provided in Supplement 1 will help facilitate this work.

## Conclusions

In this study, the COVID-19 pandemic was associated with a significant decrease in cancer incidence in Manitoba, Canada, in May and April 2020, primarily in breast, colon, and rectal cancer incidence. Breast and lung cancer incidence among individuals 75 years and older, urinary, and brain and central nervous system cancers showed sustained decreases. All other cancer sites demonstrated minimal long-term changes in incidence. However, the cumulative deficit for some high-fatality cancers is concerning and needs attention from health care delivery organizations. Because of the heterogeneity in the association between the COVID-19 pandemic and cancer incidence, observational studies should be done to examine this association in other regions.

## Notes

Supplement 1. **eTable 1.** Description of cancer cases diagnosed in Manitoba, Canada from January 2015 to December 2021 **eTable 2.** Ratios and 95% confidence intervals between fitted and counterfactual incidence values for cancer sites with no significant COVID-19-by-time interaction from April 2020 to December 2021 **eTable 3.** Ratios and 95% confidence intervals between fitted and counterfactual incidence values for cancer sites with a significant COVID-19-by-time interaction by month from April 2020 to December 2021 **eTable 4.** Ratios and 95% confidence intervals between fitted and counterfactual incidence values for breast, colon, rectal, and lung cancer by age group and month from April 2020 to December 2021 **eTable 5.** Estimated cumulative fitted and counterfactual number of cancer cases, difference, and percent difference by site as of December 31, 2021 **eFigure 1.** Incidence rate for breast cancer for women <50, 50 to 74, and ≥75 years of age by month, Manitoba **eFigure 2.** Incidence rate for lung cancer for individuals <50 years, 50 to 74, and ≥75 years of age by month, Manitoba **eFigure 3.** Incidence rate for colon cancer for individuals <50, 50 to 74, and ≥75 years of age by month, Manitoba **eFigure 4.** Incidence rate for rectal cancer for individuals <50 years of age, 50 to 74, and ≥75 years of age by month, Manitoba **eAppendix 1.** Cancer types included in each category and International Classification of Diseases for Oncology version 3 (ICD-O-3) codes **eAppendix 2.** R code and simulated data for an interrupted time series analysis

Supplement 2.
Data Sharing Statement
