# Supplementary material for: New Cancer Diagnoses Before and During the COVID-19 Pandemic
Source: JAMA Netw Open. 2023 Sep 5;6(9):e2332363. doi: 10.1001/jamanetworkopen.2023.32363 (PMC10481240; doi:10.1001/jamanetworkopen.2023.32363)
Supplement: Supplement 1. — eTable 1. Description of cancer cases diagnosed in Manitoba, Canada from January 2015 to December 2021 eTable 2. Ratios and 95% confidence intervals between fitted and counterfactual incidence values for cancer sites with no significant COVID-19-by-time interaction from April 2020 to December 2021 eTable 3. Ratios and 95% confidence intervals between fitted and counterfactual incidence values for cancer sites with a significant COVID-19-by-time interaction by month from April 2020 to December 2021 eTable 4. Ratios and 95% confidence intervals between fitted and counterfactual incidence values for breast, colon, rectal, and lung cancer by age group and month from April 2020 to December 2021 eTable 5. Estimated cumulative fitted and counterfactual number of cancer cases, difference, and percent difference by site as of December 31, 2021 eFigure 1. Incidence rate for breast cancer for women <50, 50 to 74, and ≥75 years of age by month, Manitoba eFigure 2. Incidence rate for lung cancer for individuals <50 years, 50 to 74, and ≥75 years of age by month, Manitoba eFigure 3. Incidence rate for colon cancer for individuals <50, 50 to 74, and ≥75 years of age by month, Manitoba eFigure 4. Incidence rate for rectal cancer for individuals <50 years of age, 50 to 74, and ≥75 years of age by month, Manitoba eAppendix 1. Cancer types included in each category and International Classification of Diseases for Oncology version 3 (ICD-O-3) codes eAppendix 2. R code and simulated data for an interrupted time series analysis [file jamanetwopen-e2332363-s001.pdf]

Supplemental Online Content

Decker KM, Feely A, Bucher O, et al. New cancer diagnoses before and during the COVID-19 pandemic. *JAMA Netw Open*. 2023;6(9):e2332363. doi:10.1001/jamanetworkopen.2023.32363

eTable 1. Description of cancer cases diagnosed in Manitoba, Canada from January 2015 to December 2021

eTable 2. Ratios and 95% confidence intervals between fitted and counterfactual incidence values for cancer sites with no significant COVID-19-by-time interaction from April 2020 to December 2021

eTable 3. Ratios and 95% confidence intervals between fitted and counterfactual incidence values for cancer sites with a significant COVID-19-by-time interaction by month from April 2020 to December 2021

eTable 4. Ratios and 95% confidence intervals between fitted and counterfactual incidence values for breast, colon, rectal, and lung cancer by age group and month from April 2020 to December 2021

eTable 5. Estimated cumulative fitted and counterfactual number of cancer cases, difference, and percent difference by site as of December 31, 2021

eFigure 1. Incidence rate for breast cancer for women <50, 50 to 74, and ≥75 years of age by month, Manitoba

eFigure 2. Incidence rate for lung cancer for individuals <50 years, 50 to 74, and ≥75 years of age by month, Manitoba

eFigure 3. Incidence rate for colon cancer for individuals <50, 50 to 74, and ≥75 years of age by month, Manitoba

eFigure 4. incidence rate for rectal cancer for individuals <50 years of age, 50 to 74, and ≥75 years of age by month, Manitob

eAppendix 1. Cancer types included in each category and International Classification of Diseases for Oncology version 3 (ICD-O-3) codes

eAppendix 2. R code and simulated data for an interrupted time series analysis

This supplemental material has been provided by the authors to give readers additional information about their work.

© 2023 Decker KM et al. *JAMA Network Open.*

eTable 1. Description of cancer cases diagnosed in Manitoba, Canada from January 2015 to December 2021

Cancer site	Number of cases	Median age at diagnosis	Sex, n (%)	
			Male	Female
**All**	48,378	68	24,404 (50.4)	23,972 (49.6)
**Breast**	6,548	65	0 (0.0)	6,548 (100.0)
**Lung**	6,477	72	3,102 (47.9)	3,375 (52.1)
**Prostate**	5,849	69	5,849 (100.0)	0 (0.0)
**Colon**	3,876	71	1,985 (51.2)	1,891 (48.8)
**Rectal**	2,012	66	1,273 (63.3)	739 (36.7)
**Hematologic**	4,655	68	2,678 (57.5)	1,977 (42.5)
**Unknown primary**	1,764	74	941 (53.3)	823 (46.7)
**Head and neck**	1,674	66	1,203 (71.9)	471 (28.1)
**Endocrine**	1,130	52	325 (28.8)	805 (71.2)
**Melanoma**	1,952	67	1,095 (56.1)	857 (43.9)
**Brain and central nervous system**	662	61	384 (58.0)	278 (42.0)
**Urinary**	2,982	69	2,120 (71.1)	862 (28.9)
**Gynecologic**	3,228	63	0 (0.0)	3,228 (100.0)
**Other digestive**	3,164	69	1,966 (62.1)	1,198 (37.9)
**Pancreatic**	1,352	72	683 (50.5)	669 (49.5)
**Other**	1,053	58	800 (76.0)	251 (23.8)

© 2023 Decker KM et al. *JAMA Network Open.*

eTable 2. Ratios and 95% confidence intervals between fitted and counterfactual incidence values for cancer sites with no significant COVID-19-by-time interaction from April 2020 to December 2021

Time frame	Prostate	Gynecological	Other digestive	Pancreas	Brain and central nervous system	Other	Urinary
**Apr 2020 to Dec 2021**	0.94 (0.83, 1.07)	1.01 (0.90, 1.13)	0.99 (0.88, 1.11)	1.07 (0.89, 1.29)	0.74 (0.58, 0.96)	1.10 (0.90, 1.35)	0.88 (0.78, 1.00)

© 2023 Decker KM et al. *JAMA Network Open.*

eTable 3. Ratios and 95% confidence intervals between fitted and counterfactual incidence values for cancer sites with a significant COVID-19-by-time interaction by month from April 2020 to December 2021

Cancer Site	All	Breast	Lung	Colon	Rectal	Hematologic	Unknown primary	Head and neck	Endocrine	Melanoma
**2020**										
**Apr**	0.77 (0.70, 0.84)	0.54 (0.42, 0.68)	0.98 (0.88, 1.08)	0.65 (0.54, 0.78)	0.53 (0.33, 0.84)	1.12 (1.00, 1.26)	1.08 (0.87, 1.35)	0.50 (0.34, 0.74)	0.77 (0.56, 1.05)	0.35 (0.18, 0.66)
**May**	0.77 (0.70, 0.84)	0.54 (0.42, 0.68)	0.98 (0.88, 1.08)	0.65 (0.54, 0.78)	1.05 (0.79, 1.38)	1.12 (1.00, 1.26)	1.08 (0.87, 1.35)	0.50 (0.34, 0.74)	0.79 (0.58, 1.06)	0.91 (0.77, 1.08)
**Jun**	0.97 (0.93, 1.01)	0.89 (0.80, 0.99)	0.98 (0.88, 1.08)	0.65 (0.54, 0.78)	1.05 (0.79, 1.38)	1.12 (1.00, 1.26)	1.08 (0.87, 1.35)	1.00 (0.85, 1.16)	0.81 (0.61, 1.08)	0.91 (0.77, 1.08)
**Jul**	0.97 (0.93, 1.01)	0.89 (0.80, 0.99)	0.98 (0.88, 1.08)	0.65 (0.54, 0.78)	1.05 (0.79, 1.38)	1.12 (1.00, 1.26)	1.08 (0.87, 1.35)	1.00 (0.85, 1.16)	0.83 (0.63, 1.09)	0.91 (0.77, 1.08)
**Aug**	0.97 (0.93, 1.01)	0.89 (0.80, 0.99)	0.98 (0.88, 1.08)	0.93 (0.83, 1.04)	1.05 (0.79, 1.38)	1.12 (1.00, 1.26)	1.08 (0.87, 1.35)	1.00 (0.85, 1.16)	0.85 (0.65, 1.11)	0.91 (0.77, 1.08)
**Sep**	0.97 (0.93, 1.01)	0.89 (0.80, 0.99)	0.98 (0.88, 1.08)	0.93 (0.83, 1.04)	1.05 (0.79, 1.38)	1.12 (1.00, 1.26)	1.08 (0.87, 1.35)	1.00 (0.85, 1.16)	0.87 (0.67, 1.12)	0.91 (0.77, 1.08)
**Oct**	0.97 (0.93, 1.01)	0.89 (0.80, 0.99)	0.98 (0.88, 1.08)	0.93 (0.83, 1.04)	1.05 (0.79, 1.38)	1.12 (1.00, 1.26)	1.08 (0.87, 1.35)	1.00 (0.85, 1.16)	0.89 (0.70, 1.15)	0.91 (0.77, 1.08)
**Nov**	0.97 (0.93, 1.01)	0.89 (0.80, 0.99)	0.98 (0.88, 1.08)	0.93 (0.83, 1.04)	1.05 (0.79, 1.38)	1.12 (1.00, 1.26)	1.08 (0.87, 1.35)	1.00 (0.85, 1.16)	0.91 (0.72, 1.17)	0.91 (0.77, 1.08)
**Dec**	0.97 (0.93, 1.01)	0.89 (0.80, 0.99)	0.89 (0.81, 0.99)	0.93 (0.83, 1.04)	1.05 (0.79, 1.38)	1.12 (1.00, 1.26)	1.08 (0.87, 1.35)	1.00 (0.85, 1.16)	0.94 (0.74, 1.19)	0.91 (0.77, 1.08)
**2021**										
**Jan**	0.97 (0.93, 1.01)	0.89 (0.80, 0.99)	0.89 (0.81, 0.99)	0.93 (0.83, 1.04)	1.05 (0.79, 1.38)	0.96 (0.85, 1.09)	0.82 (0.65, 1.03)	1.00 (0.85, 1.16)	0.96 (0.76, 1.22)	0.91 (0.77, 1.08)
**Feb**	0.97 (0.93, 1.01)	0.89 (0.80, 0.99)	0.89 (0.81, 0.99)	0.93 (0.83, 1.04)	1.05 (0.79, 1.38)	0.96 (0.85, 1.09)	0.82 (0.65, 1.03)	1.00 (0.85, 1.16)	0.99 (0.77, 1.25)	0.91 (0.77, 1.08)
**Mar**	0.97 (0.93, 1.01)	0.89 (0.80, 0.99)	0.89 (0.81, 0.99)	0.93 (0.83, 1.04)	1.05 (0.79, 1.38)	0.96 (0.85, 1.09)	0.82 (0.65, 1.03)	1.00 (0.85, 1.16)	1.01 (0.79, 1.29)	0.91 (0.77, 1.08)
**Apr**	0.97 (0.93, 1.01)	0.89 (0.80, 0.99)	0.89 (0.81, 0.99)	0.93 (0.83, 1.04)	1.05 (0.79, 1.38)	0.96 (0.85, 1.09)	0.82 (0.65, 1.03)	1.00 (0.85, 1.16)	1.04 (0.81, 1.33)	0.91 (0.77, 1.08)
**May**	0.97 (0.93, 1.01)	0.89 (0.80, 0.99)	0.89 (0.81, 0.99)	0.93 (0.83, 1.04)	1.05 (0.79, 1.38)	0.96 (0.85, 1.09)	0.82 (0.65, 1.03)	1.00 (0.85, 1.16)	1.06 (0.82, 1.37)	0.91 (0.77, 1.08)

© 2023 Decker KM et al. *JAMA Network Open.*

**Jun**	0.97 (0.93, 1.01)	0.89 (0.80, 0.99)	0.89 (0.81, 0.99)	0.93 (0.83, 1.04)	1.05 (0.79, 1.38)	0.96 (0.85, 1.09)	0.82 (0.65, 1.03)	1.00 (0.85, 1.16)	1.09 (0.84, 1.42)	0.91 (0.77, 1.08)
**Jul**	0.97 (0.93, 1.01)	0.89 (0.80, 0.99)	0.89 (0.81, 0.99)	0.93 (0.83, 1.04)	1.05 (0.79, 1.38)	0.96 (0.85, 1.09)	0.82 (0.65, 1.03)	1.00 (0.85, 1.16)	1.12 (0.85, 1.47)	0.91 (0.77, 1.08)
**Aug**	0.97 (0.93, 1.01)	0.89 (0.80, 0.99)	0.89 (0.81, 0.99)	0.93 (0.83, 1.04)	1.05 (0.79, 1.38)	0.96 (0.85, 1.09)	0.82 (0.65, 1.03)	1.00 (0.85, 1.16)	1.15 (0.86, 1.52)	0.91 (0.77, 1.08)
**Sep**	0.97 (0.93, 1.01)	0.89 (0.80, 0.99)	0.89 (0.81, 0.99)	0.93 (0.83, 1.04)	1.05 (0.79, 1.38)	0.96 (0.85, 1.09)	0.82 (0.65, 1.03)	1.00 (0.85, 1.16)	1.17 (0.87, 1.58)	0.91 (0.77, 1.08)
**Oct**	0.97 (0.93, 1.01)	0.89 (0.80, 0.99)	0.89 (0.81, 0.99)	0.93 (0.83, 1.04)	1.05 (0.79, 1.38)	0.96 (0.85, 1.09)	0.82 (0.65, 1.03)	1.00 (0.85, 1.16)	1.20 (0.88, 1.64)	0.91 (0.77, 1.08)
**Nov**	0.97 (0.93, 1.01)	0.89 (0.80, 0.99)	0.89 (0.81, 0.99)	0.93 (0.83, 1.04)	1.05 (0.79, 1.38)	0.96 (0.85, 1.09)	0.82 (0.65, 1.03)	1.00 (0.85, 1.16)	1.23 (0.89, 1.71)	0.91 (0.77, 1.08)
**Dec**	0.97 (0.93, 1.01)	0.89 (0.80, 0.99)	0.89 (0.81, 0.99)	0.93 (0.83, 1.04)	1.05 (0.79, 1.38)	0.96 (0.85, 1.09)	0.82 (0.65, 1.03)	1.00 (0.85, 1.16)	1.27 (0.90, 1.78)	0.91 (0.77, 1.08)

© 2023 Decker KM et al. *JAMA Network Open.*

eTable 4. Ratios and 95% confidence intervals between fitted and counterfactual incidence values for breast, colon, rectal, and lung cancer by age group and month from April 2020 to December 2021

Cancer Site	Breast			Colon			Rectal			Lung		
**Age Group**	**<50**	**50-74**	**75+**	**<50**	**50-74**	**75+**	**<50**	**50-74**	**75+**	**<50**	**50-74**	**75+**
**2020**												
**Apr**	1.03 (0.81, 1.31)	0.54 (0.36, 0.80)	0.80 (0.65, 0.97)	0.91 (0.57, 1.45)	0.66 (0.52, 0.83)	0.66 (0.51, 0.85)	1.21 (0.81, 1.80)	0.55 (0.32, 0.92)	1.21 (0.86, 1.72)	1.21 (0.65, 2.24)	1.02 (0.91, 1.14)	0.54 (0.40, 0.73)
**May**	1.03 (0.81, 1.31)	0.27 (0.16, 0.46)	0.80 (0.65, 0.97)	0.91 (0.57, 1.45)	0.66 (0.52, 0.83)	0.66 (0.51, 0.85)	1.21 (0.81, 1.80)	1.16 (0.95, 1.42)	1.21 (0.86, 1.72)	1.21 (0.65, 2.24)	1.02 (0.91, 1.14)	0.75 (0.61, 0.92)
**Jun**	1.03 (0.81, 1.31)	0.86 (0.73, 1.01)	0.80 (0.65, 0.97)	0.91 (0.57, 1.45)	0.66 (0.52, 0.83)	0.66 (0.51, 0.85)	1.21 (0.81, 1.80)	1.16 (0.95, 1.42)	1.21 (0.86, 1.72)	1.21 (0.65, 2.24)	1.02 (0.91, 1.14)	1.04 (0.85, 1.28)
**Jul**	1.03 (0.81, 1.31)	0.86 (0.73, 1.00)	0.80 (0.65, 0.97)	0.91 (0.57, 1.45)	0.66 (0.52, 0.83)	0.66 (0.51, 0.85)	1.21 (0.81, 1.80)	1.16 (0.95, 1.42)	1.21 (0.86, 1.72)	1.21 (0.65, 2.24)	1.02 (0.91, 1.14)	1.45 (1.07, 1.95)
**Aug**	1.03 (0.81, 1.31)	0.86 (0.74, 1.00)	0.80 (0.65, 0.97)	0.91 (0.57, 1.45)	0.66 (0.52, 0.83)	0.97 (0.83, 1.14)	1.21 (0.81, 1.80)	1.16 (0.95, 1.42)	1.21 (0.86, 1.72)	1.21 (0.65, 2.24)	1.02 (0.91, 1.14)	0.72 (0.60, 0.87)
**Sep**	1.03 (0.81, 1.31)r	0.86 (0.75, 0.99)	0.80 (0.65, 0.97)	0.91 (0.57, 1.45)	0.92 (0.78, 1.09)	0.97 (0.83, 1.14)	1.21 (0.81, 1.80)	1.16 (0.95, 1.42)	1.21 (0.86, 1.72)	1.21 (0.65, 2.24)	1.02 (0.91, 1.14)	0.73 (0.61, 0.87)
**Oct**	1.03 (0.81, 1.31)	0.86 (0.75, 0.99)	0.80 (0.65, 0.97)	0.91 (0.57, 1.45)	0.92 (0.78, 1.09)	0.97 (0.83, 1.14)	1.21 (0.81, 1.80)	1.16 (0.95, 1.42)	1.21 (0.86, 1.72)	1.21 (0.65, 2.24)	1.02 (0.91, 1.14)	0.73 (0.62, 0.87)
**Nov**	1.03 (0.81, 1.31)	0.86 (0.76, 0.98)	0.80 (0.65, 0.97)	0.91 (0.57, 1.45)	0.92 (0.78, 1.09)	0.97 (0.83, 1.14)	1.21 (0.81, 1.80)	1.16 (0.95, 1.42)	1.21 (0.86, 1.72)	1.21 (0.65, 2.24)	1.02 (0.91, 1.14)	0.74 (0.63, 0.87)
**Dec**	1.03 (0.81, 1.31)	0.86 (0.76, 0.98)	0.80 (0.65, 0.97)	0.91 (0.57, 1.45)	0.92 (0.78, 1.09)	0.97 (0.83, 1.14)	1.21 (0.81, 1.80)	1.16 (0.95, 1.42)	1.21 (0.86, 1.72)	1.21 (0.65, 2.24)	1.02 (0.91, 1.14)	0.75 (0.64, 0.87)

© 2023 Decker KM et al. *JAMA Network Open.*

**2021**												
**Jan**	1.03 (0.81, 1.31)	0.86 (0.76, 0.98)	0.80 (0.65, 0.97)	0.91 (0.57, 1.45)	0.92 (0.78, 1.09)	0.97 (0.83, 1.14)	1.21 (0.81, 1.80)	1.16 (0.95, 1.42)	1.21 (0.86, 1.72)	1.21 (0.65, 2.24)	1.02 (0.91, 1.14)	0.75 (0.65, 0.87)
**Feb**	1.03 (0.81, 1.31)	0.87 (0.76, 0.98)	0.80 (0.65, 0.97)	0.91 (0.57, 1.45)	0.92 (0.78, 1.09)	0.97 (0.83, 1.14)	1.21 (0.81, 1.80)	1.16 (0.95, 1.42)	1.21 (0.86, 1.72)	1.21 (0.65, 2.24)	1.02 (0.91, 1.14)	0.76 (0.66, 0.88)
**Mar**	1.03 (0.81, 1.31)	0.87 (0.76, 0.98)	0.80 (0.65, 0.97)	0.91 (0.57, 1.45)	0.92 (0.78, 1.09)	0.97 (0.83, 1.14)	1.21 (0.81, 1.80)	1.16 (0.95, 1.42)	1.21 (0.86, 1.72)	1.21 (0.65, 2.24)	1.02 (0.91, 1.14)	0.77 (0.67, 0.88)
**Apr**	1.03 (0.81, 1.31)	0.87 (0.76, 0.99)	0.80 (0.65, 0.97)	0.91 (0.57, 1.45)	0.92 (0.78, 1.09)	0.97 (0.83, 1.14)	1.21 (0.81, 1.80)	1.16 (0.95, 1.42)	1.21 (0.86, 1.72)	1.21 (0.65, 2.24)	1.02 (0.91, 1.14)	0.78 (0.67, 0.89)
**May**	1.03 (0.81, 1.31)	0.87 (0.76, 0.99)	0.80 (0.65, 0.97)	0.91 (0.57, 1.45)	0.92 (0.78, 1.09)	0.97 (0.83, 1.14)	1.21 (0.81, 1.80)	1.16 (0.95, 1.42)	1.21 (0.86, 1.72)	1.21 (0.65, 2.24)	1.02 (0.91, 1.14)	0.78 (0.68, 0.90)
**Jun**	1.03 (0.81, 1.31)	0.87 (0.76, 1.00)	0.80 (0.65, 0.97)	0.91 (0.57, 1.45)	0.92 (0.78, 1.09)	0.97 (0.83, 1.14)	1.21 (0.81, 1.80)	1.16 (0.95, 1.42)	1.21 (0.86, 1.72)	1.21 (0.65, 2.24)	1.02 (0.91, 1.14)	0.79 (0.68, 0.92)
**Jul**	1.03 (0.81, 1.31)	0.87 (0.76, 1.01)	0.80 (0.65, 0.97)	0.91 (0.57, 1.45)	0.92 (0.78, 1.09)	0.97 (0.83, 1.14)	1.21 (0.81, 1.80)	1.16 (0.95, 1.42)	1.21 (0.86, 1.72)	1.21 (0.65, 2.24)	1.02 (0.91, 1.14)	0.80 (0.68, 0.93)
**Aug**	1.03 (0.81, 1.31)	0.87 (0.76, 1.02)	0.80 (0.65, 0.97)	0.91 (0.57, 1.45)	0.92 (0.78, 1.09)	0.97 (0.83, 1.14)	1.21 (0.81, 1.80)	1.16 (0.95, 1.42)	1.21 (0.86, 1.72)	1.21 (0.65, 2.24)	1.02 (0.91, 1.14)	0.80 (0.68, 0.95)
**Sep**	1.03 (0.81, 1.31)	0.88 (0.74, 1.04)	0.80 (0.65, 0.97)	0.91 (0.57, 1.45)	0.92 (0.78, 1.09)	0.97 (0.83, 1.14)	1.21 (0.81, 1.80)	1.16 (0.95, 1.42)	1.21 (0.86, 1.72)	1.21 (0.65, 2.24)	1.02 (0.91, 1.14)	0.81 (0.68, 0.96)
**Oct**	1.03 (0.81, 1.31)	0.88 (0.74, 1.04)	0.80 (0.65, 0.97)	0.91 (0.57, 1.45)	0.92 (0.78, 1.09)	0.97 (0.83, 1.14)	1.21 (0.81, 1.80)	1.16 (0.95, 1.42)	1.21 (0.86, 1.72)	1.21 (0.65, 2.24)	1.02 (0.91, 1.14)	0.82 (0.68, 0.98)
**Nov**	1.03 (0.81, 1.31)	0.88 (0.74, 1.05)	0.80 (0.65, 0.97)	0.91 (0.57, 1.45)	0.92 (0.78, 1.09)	0.97 (0.83, 1.14)	1.21 (0.81, 1.80)	1.16 (0.95, 1.42)	1.21 (0.86, 1.72)	1.21 (0.65, 2.24)	1.02 (0.91, 1.14)	0.83 (0.68, 1.00)
**Dec**	1.03 (0.81, 1.31)	0.88 (0.73, 1.06)	0.80 (0.65, 0.97)	0.91 (0.57, 1.45)	0.92 (0.78, 1.09)	0.97 (0.83, 1.14)	1.21 (0.81, 1.80)	1.16 (0.95, 1.42)	1.21 (0.86, 1.72)	1.21 (0.65, 2.24)	1.02 (0.91, 1.14)	0.83 (0.68, 1.03)

© 2023 Decker KM et al. *JAMA Network Open.*

eTable 5. Estimated cumulative fitted and counterfactual number of cancer cases, difference, and percent difference by site as of December 31, 2021

Cancer site	Fitted cancer cases (n)	Counterfactual cancer cases (n)	Cumulative difference	Percent cumulative difference* (95% CI)
**Overall**	12,458	13,150	-692	-5.3 (-9.2, -1.0)
**Breast**	1,662	1,935	-273	-14.1 (-22.5, -5.8)
**Lung**	1,600	1,732	-132	-7.6 (-15.5, +0.5)
**Prostate**	1,674	1,773	-99	-5.6 (-17.2, +6.9)
**Colon**	955	1,087	-133	-12.2 (-20.9, -2.7)
**Rectal**	512	499	+12	+2.4 (-21.7, +32.6)
**Hematologic**	1155	1121	+34	+3.0 (-7.6, +14.6)
**Urinary**	744	843	-99	-11.7 (-21.9, -0.3)
**Unknown primary**	400	429	-29	-6.8 (-24.4, +11.6)
**Head and neck**	449	472	-24	-5.0 (-18.5, +9.6)
**Brain and central nervous system**	164	220	-56	-25.6 (-41.7, -4.8)
**Gynecologic**	817	809	+8	+1.0 (-9.9–12.8)
**Other digestive**	863	873	-10	-1.2 (-12.2, +10.9)
**Melanoma**	524	592	-68	-11.5 (-24.4, +4.4)
**Pancreatic**	365	340	+25	+7.3 (-12.1, +28.1)
**Endocrine**	305	305	0	0.0 (-22.7, +25.7)
**Other**	271	246	+25	+10.3 (-10.1, +35.2)

* A minus sign indicates a deficit and a plus sign indicates a surplus. Abbreviations: CI, confidence interval

© 2023 Decker KM et al. *JAMA Network Open.*

eFigure 1. Incidence rate for breast cancer for women <50, 50 to 74, and ≥75 years of age by month, Manitoba

<50 years of age

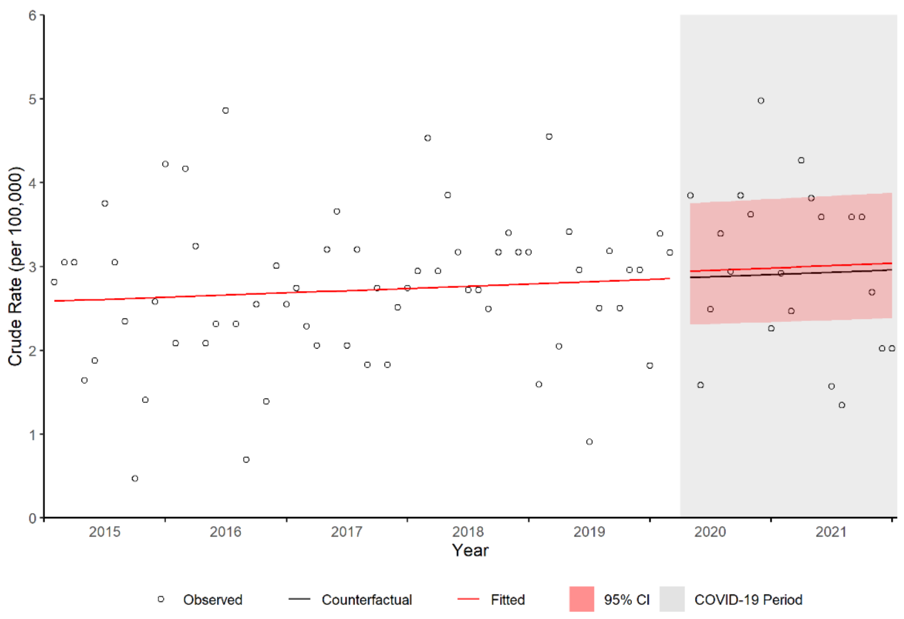

50 to 74 years of age

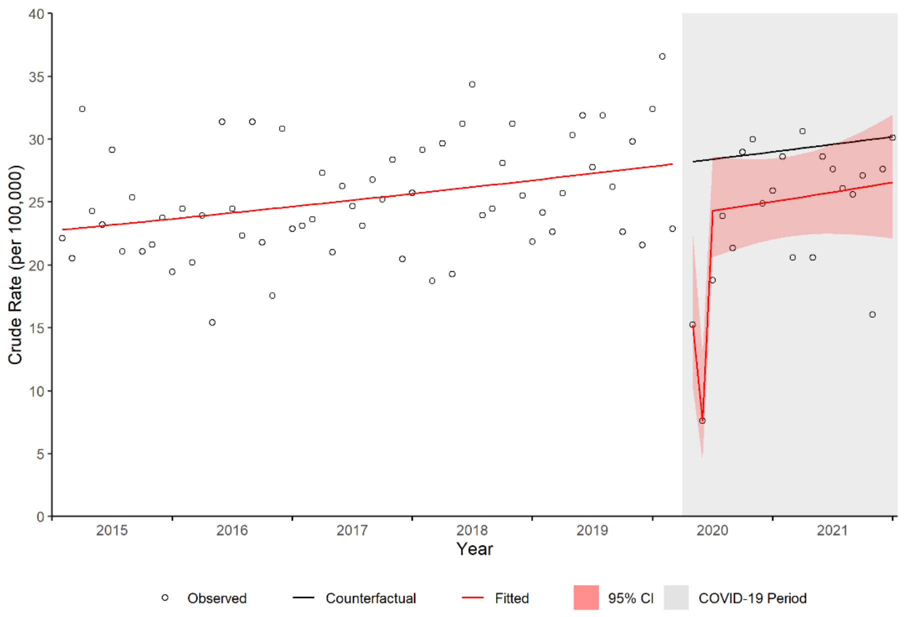

© 2023 Decker KM et al. *JAMA Network Open.*

75+ years of age

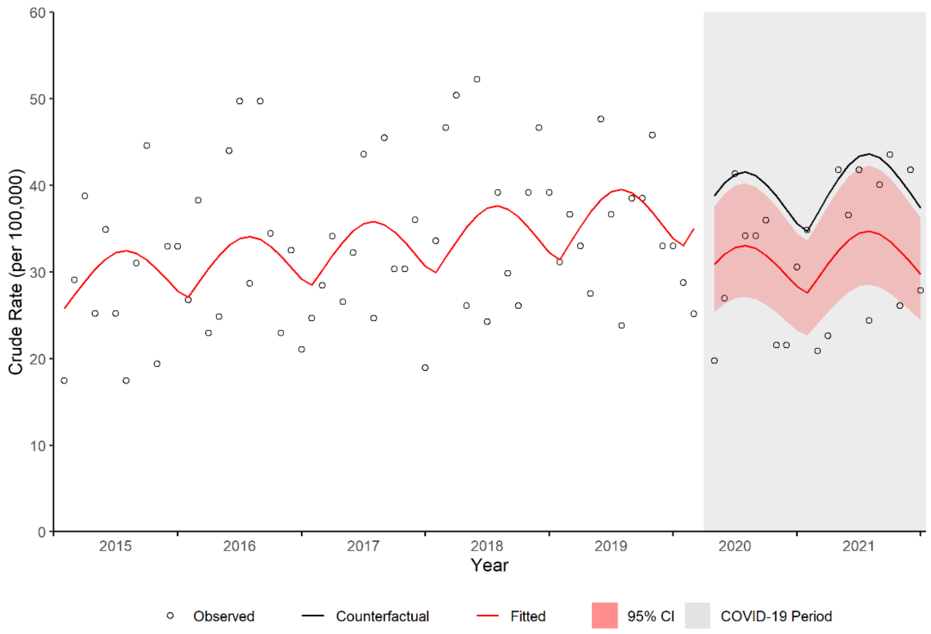

© 2023 Decker KM et al. *JAMA Network Open.*

eFigure 2. Incidence rate for lung cancer for individuals <50 years, 50 to 74, and ≥75 years of age by month, Manitoba

<50 years of age

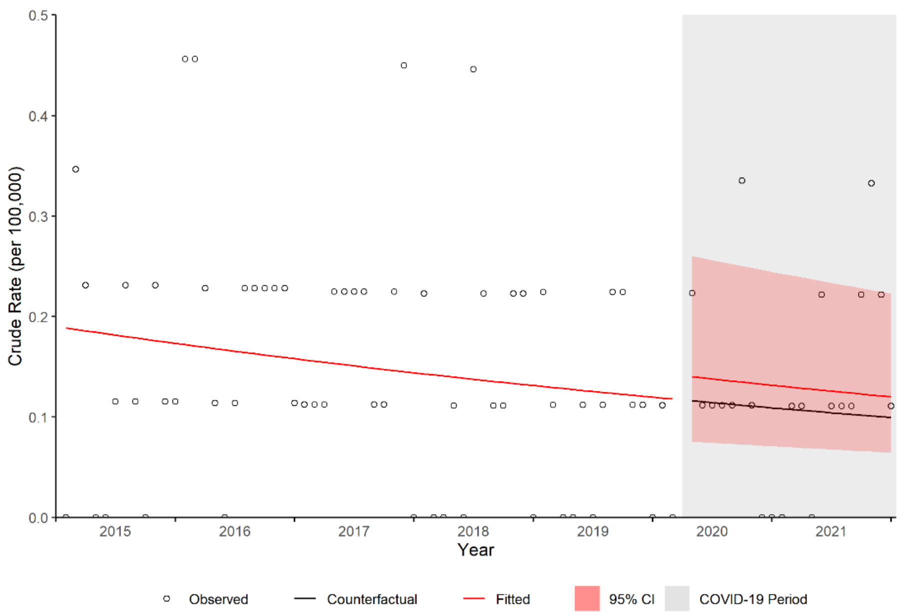

50-74 years of age

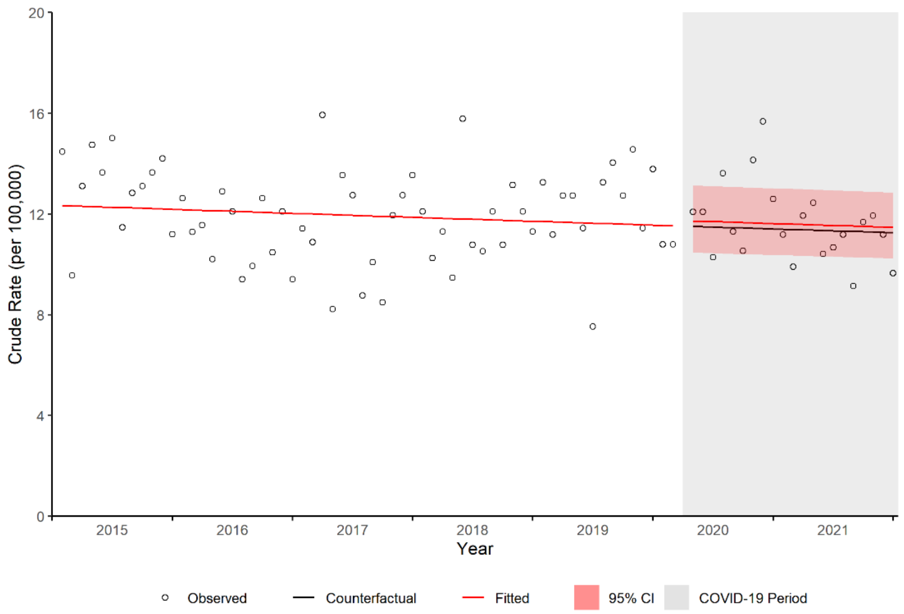

© 2023 Decker KM et al. *JAMA Network Open.*

75+ years of age

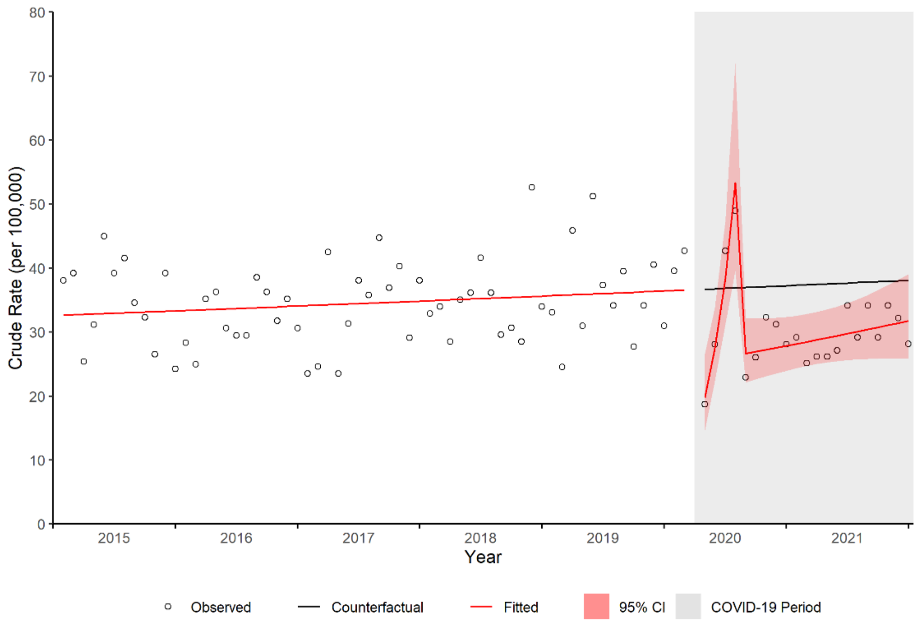

© 2023 Decker KM et al. *JAMA Network Open.*

eFigure 3. Incidence rate for colon cancer for individuals <50, 50 to 74, and ≥75 years of age by month, Manitoba

<50 years of age

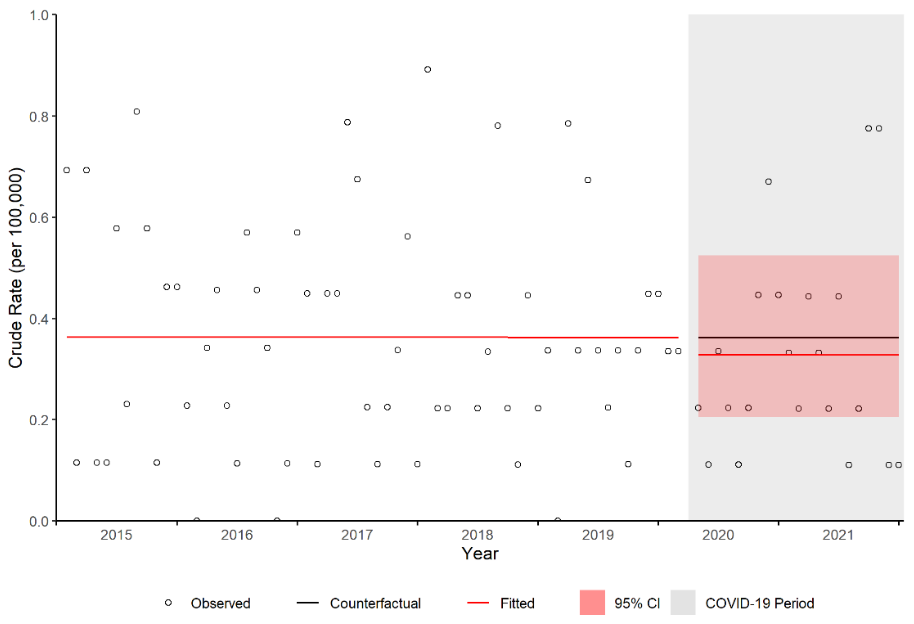

50-74 years of age

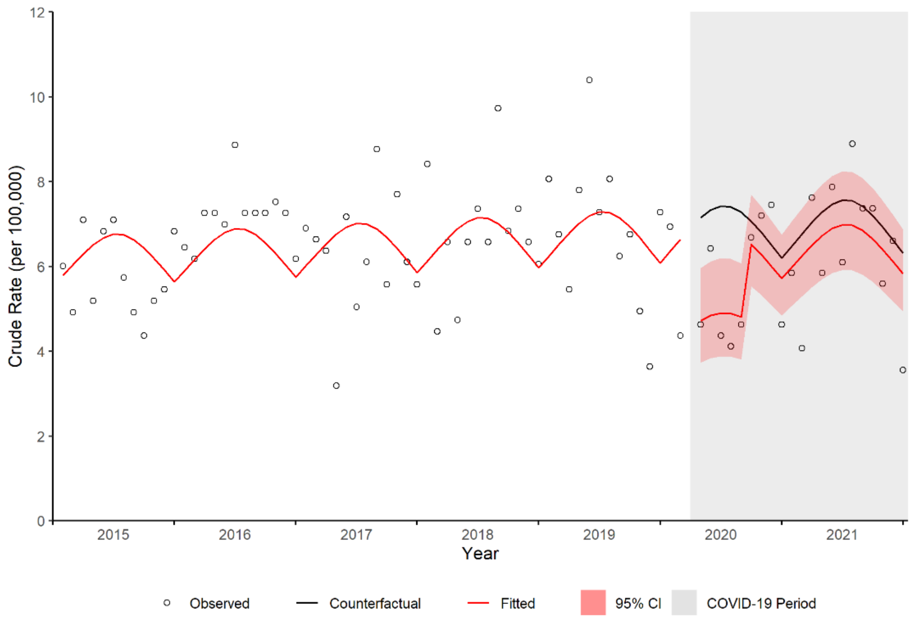

© 2023 Decker KM et al. *JAMA Network Open.*

75+ years of age

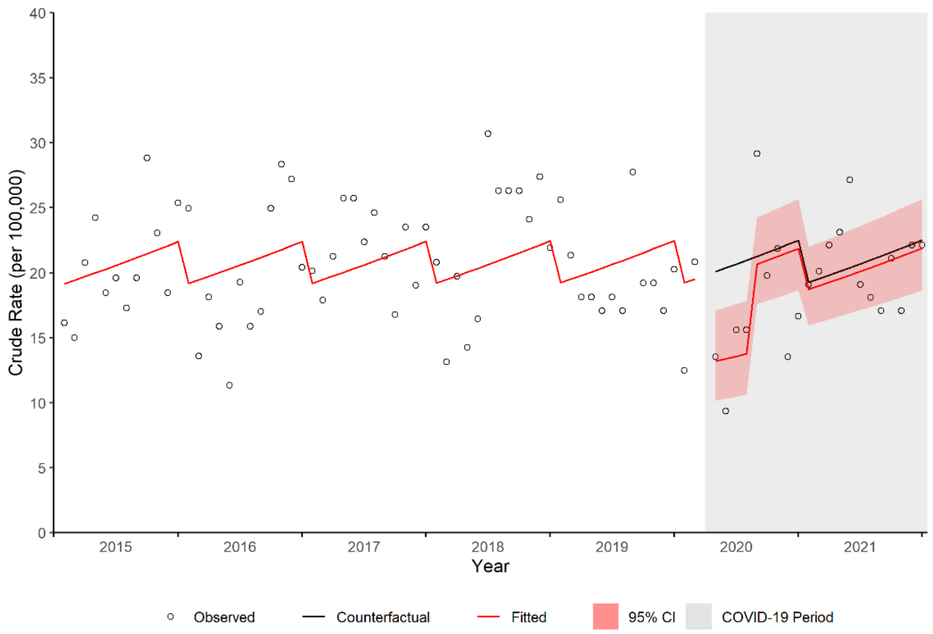

© 2023 Decker KM et al. *JAMA Network Open.*

eFigure 4. incidence rate for rectal cancer for individuals <50 years of age, 50 to 74, and ≥75 years of age by month, Manitoba

<50 years of age

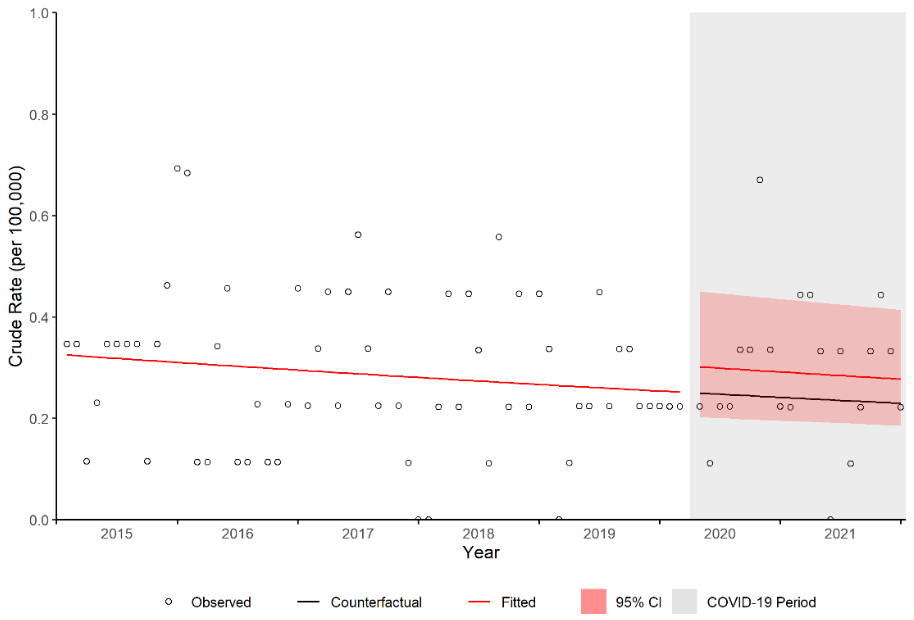

50-74 years of age

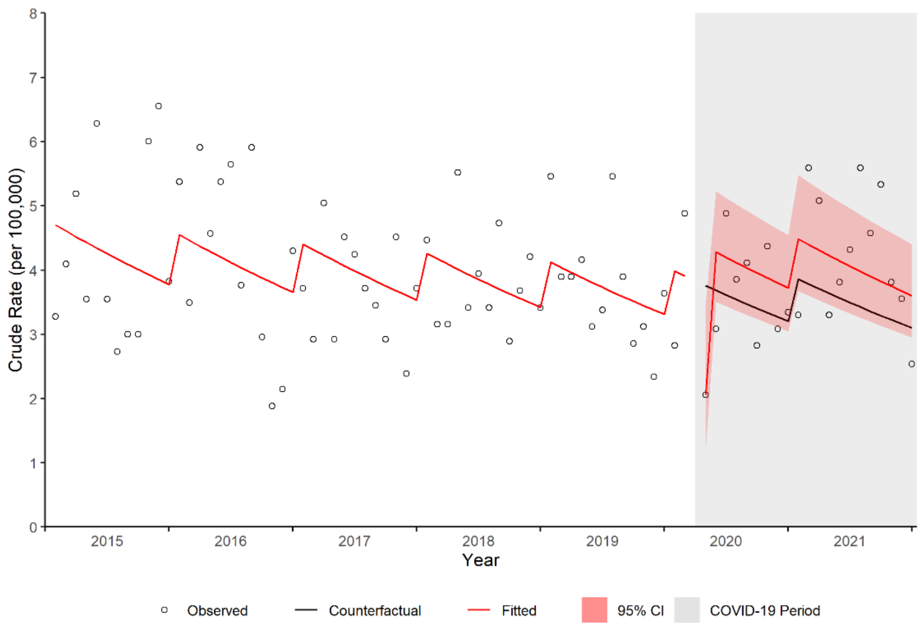

© 2023 Decker KM et al. *JAMA Network Open.*

75+ years of age

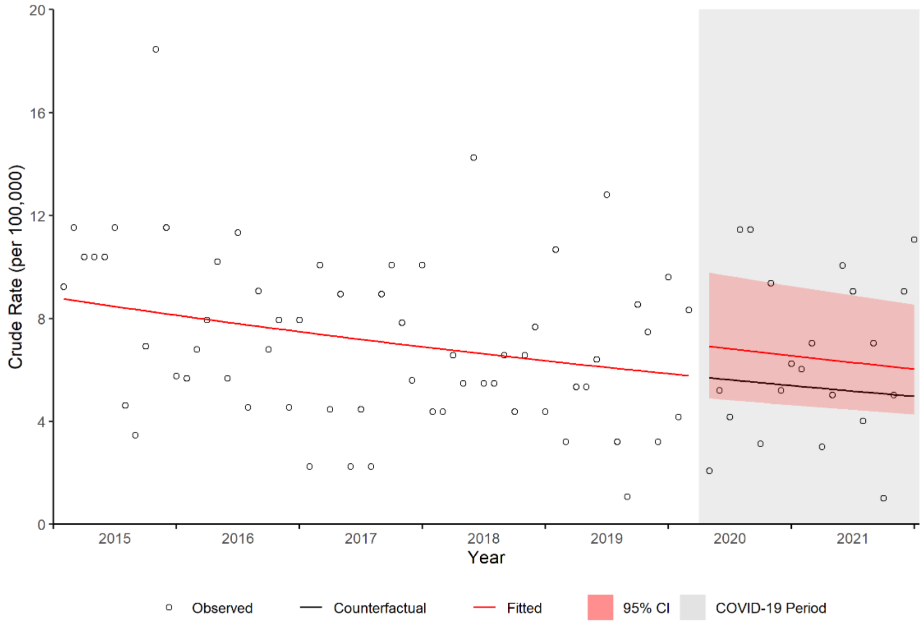

© 2023 Decker KM et al. *JAMA Network Open.*

eAppendix A. Cancer types included in each category and International Classification of Diseases for Oncology version 3 (ICD-O-3) codes

Category	Cancer sites	ICD-O-3 codes
**Breast**	Female breast	C500-C509
**Lung**	Small cell and non-small cell	C340-C349
**Prostate**	Prostate	C619
**Colon**	Colon	C180-189, C260
**Rectal**	Rectal and rectosigmoid	C199, C209
**Hematologic**	Hodgkin lymphoma	9650-9667
	Non-Hodgkin lymphoma	9590-9597, 9670-9671, 9673, 9675, 9678-9680, 9684, 9687-9691, 9695, 9698-9702, 9705, 9708-9709, 9712, 9714-9719, 9724-9729, 9735, 9737, 9738,<br>9811-9818 (except C420, C421, C424)<br>9823 (except C420, C421, C424)<br>9827 (except C420, C421, C424)<br>9837 (except C420, C421, C424)
	Myeloma	9731-9732, 9734
	Acute lymphocytic leukemia	9826, 9835-9836<br>9811-9818 (only C421)<br>9837 (only C421)
	Chronic lymphocytic leukemia	9823 (only C421, if SLL C77)
	Acute myeloid leukemia	9840, 9861, 9865-9867, 9869, 9871-9874, 9895-9897, 9898, 9910-9911, 9920
	Acute monocytic leukemia	9891
	Chronic myeloid leukemia	9863, 9875-9876, 9945-9946
	Other leukemia	9733, 9742, 9800, 9801, 9805-9809, 9820, 9831-9834, 9860, 9870, 9930, 9931, 9940, 9948, 9963, 9964<br>9827 (only C421)
**Urinary**	Bladder	C670-C679
	Kidney and renal pelvis	C649, C659
	Ureter	C669
	Other urinary system	C680-689
**Unknown primary**	Unknown primary	9740-9741, 9750-9769, 9950, 9960-9962, 9965-9967, 9970-9971, 9975, 9980, 9982-9987, 9989,9991-9992,<br>C420-C424 (except 9050-9055, 9140, 9590-9992)<br>C760-C768 (except 9050-9055, 9140, 9590-9992)<br>C770-C779 (except 9050-9055, 9140, 9590-9992)<br>C809 (except 9050-9055, 9140, 9590-9992)
**Head and neck**	Buccal cavity and pharynx	C00-C14
	Larynx	C320-C329
	Other non-lung respiratory	C300-C301, C310-C319, C384, C339, C381-C383, C388, C390-C399
**Brain and central nervous system**	Brain	C710-C719
	Other nervous system	C710-C719 (953), C700-C709, C720-C729
**Gynecologic**	Cervix uteri	C530-C539
	Corpus uteri	C540-C549
	Uterus, NOS	C559
	Ovary	C569
	Other female genital system	C529, C510-C519, C570-C589
**Category**	**Cancer sites**	**ICD-O-3 codes**
**Other digestive**	Esophagus	C150-C159
	Stomach	C160-C169

© 2023 Decker KM et al. *JAMA Network Open.*

	Small intestine	C170-C179
	Anus	C210-C212, C218
	Liver	C220
	Gallbladder	C239
	Other digestive system	C240-C249, C221, C480, C481-C482, C268-C269, C488
**Melanoma**	Melanoma	C440-C449 (8720-8790)
**Pancreatic**	Pancreatic	C250-C259
**Endocrine**	Thyroid	C739
	Other endocrine	C379, C740-C749, C750-C759
**Other**	Bones and joints	C400-C419
	Soft tissue (including heart)	C380, C470-C479, C490-C499
	Mesothelioma	9050-9055
	Kaposi sarcoma	9140
	Eye	C690-C699
	Male breast	C500-C509
	Testis	C620-C629
	Penis	C600-C609
	Other male genital system	C630-C639

© 2023 Decker KM et al. *JAMA Network Open.*

eAppendix B. R code and simulated data for an interrupted time series analysis

```
#######################################
###        Load packages
######################################
library(haven)
library(splines)
library(Hmisc)
library(MASS)
library(lattice)
library(DHARMa)
library(ggplot2)
library(car)
library(multcomp)
library(lmtest)
library(glmmTMB)



###########################################
###       Simulated dataset
###########################################

# This simulated dataset contains the following variables:
#   - count:             number of cases
#   - stdpop:             age-standardized population
#   - stdrate:            age-standardized incidence rate per 100,000
#   - year:               year of diagnosis
#   - month:              calendar month (for seasonality)
#   - time:               time since beginning of study period (Jan 2015)
#   - covid:              COVID restriction indicator (0=before March 2020, 1=after March 2020)

count   <-  c(523, 539, 489, 537, 589, 596, 506, 484, 547, 564, 570, 488,
                 560, 546, 504, 539, 525, 577, 555, 514, 587, 596, 597, 552,
                 565, 552, 525, 596, 611, 605, 550, 510, 531, 560, 597, 563,
                 604, 557, 553, 559, 589, 578, 606, 533, 524, 606, 569, 501,
                 576, 589, 574, 626, 598, 577, 599, 520, 617, 614, 649, 591,
                 619, 634,  NA, 432, 485, 642, 629, 574, 623, 594, 571, 552,
                 633, 569, 558, 609, 635, 616, 545, 539)
stdpop  <- c(1298008, 1303355, 1305184, 1301240, 1298734, 1299299, 1295466, 1292982, 1303310, 1297892,
1297353, 1290947,
                  1319253, 1317550, 1322917, 1322676, 1323532, 1321520, 1325732, 1329176, 1331195, 1329771,
1324808, 1323016,
                  1348566, 1346798, 1352188, 1346474, 1348969, 1350947, 1356338, 1359991, 1351885, 1354543,
1352257, 1351977,
                  1375947, 1376385, 1377656, 1379068, 1380944, 1372174, 1385761, 1379780, 1378726, 1388695,
1379005, 1384956,
                  1413056, 1396460, 1406157, 1401348, 1411815, 1410403, 1403214, 1410865, 1417457, 1410548,
1402133, 1414184,
                  1428629, 1424773,      NA, 1437775, 1440587, 1450485, 1435894, 1436122, 1440942, 1431486,
1428909, 1441184,
                  1468096, 1454458, 1455076, 1479103, 1471497, 1484575, 1492564, 1480314)
stdrate<-(count/stdpop)*100000
year  <- c(rep(2015,12),rep(2016,12),rep(2017,12),rep(2018,12),rep(2019,12),rep(2020,12),rep(2021,8))
month <- c(rep(1:12, times=6), c(1:8))
time  <- c(1:80)
covid <- c(rep(0,62), NA, rep(1,17))

inc<-as.data.frame(cbind(count,stdpop,stdrate,year,month,time,covid))


## simple plot indicates sharp drop at the beginning of the pandemic
```

© 2023 Decker KM et al. *JAMA Network Open.*

```
plot(inc$time,inc$stdrate, ylim=c(0,60))


# incidence dataset with March 2020 removed - needed for predictions & mean-variance plot
inc2 <- subset(inc, !(year==2020 & month==3))


# dataset to plot counterfactual (need covid = 0 for all months)
inc_counter <- data.frame(covid=0,
                          stdpop=inc$stdpop,
                          time=inc$time,
                          month=inc$month,
                          covid1=0,
                          covid2=0)

# add dummy covid period variables for better time interactions (see earlier plot)
inc$covid1 <- with(inc,ifelse(year==2020 & month %in% 4:5, 1, 0))
inc$covid2 <- with(inc,ifelse((year==2020 & month %in% 6:12) | year==2021, 1, 0))



###########################################
###    Diagnostics
###########################################

### Mean-Variance Plot to determine appropriate GLM model

# Poisson model
pois <- glm(count ~ offset(log(stdpop)) + covid1*time + covid2*time,
            family='poisson', data=inc)

# Negative binomial (NB1)
nb1<-glmmTMB(count ~ offset(log(stdpop)) + covid1*time + covid2*time,
             family=nbinom1,  data=inc)
out_nb1<-summary(nb1)

# Negative binomial model (NB2)
nb2 <- glm.nb(count ~ offset(log(stdpop)) + covid1*time + covid2*time,
              data=inc)

# gamma regression
gamma_log <- glm(count ~ offset(log(stdpop)) + covid1*time + covid2*time,
                 data = inc, family=Gamma(link='log'))
out_gamma <- summary(gamma_log)

# inverse gaussian
inv_gauss <- glm(count ~ offset(log(stdpop)) + covid1*time + covid2*time,
                 data = inc, family='inverse.gaussian'(link='log'))
out_inv_gauss <- summary(inv_gauss)

# generalized Poisson
gen_pois<-glmmTMB(count ~ offset(log(stdpop)) + covid1*time + covid2*time,
             family=genpois,  data=inc)
out_gen_pois<-summary(gen_pois)



####  plotting mean-variance relationship

xb <- predict(pois)  # linear predictor
g <- cut2(xb, g=5) # cut linear predictor into multiple equally-sized groups (in this case, 5 groups)
m <- tapply(inc2$count, g, mean)
```

© 2023 Decker KM et al. *JAMA Network Open.*

```
v <- tapply(inc2$count, g, var)

# Mean-Variance Relationship plot, which does not provide a clearly appropriate distribution
plot(m, v,
     xlab='Mean',
     ylab='Variance',
     main='Mean-Variance Relationship',
     xlim=c(500,650),
     ylim=c(0,3000))

x <- seq(500,650,1)
lines(x, x, col='black')                                    # Poisson model
lines(x, x*(1+out_nb1$sigma), col='blue')                   # NB1 model
lines(x, x*(1+x/nb2$theta), col='red')                      # NB2 model
lines(x, (x^2)*(out_gamma$dispersion), col='green')         # gamma (log) model
lines(x, (x^3)*(out_inv_gauss$dispersion), col='magenta')   # gaussian inverse
lines(x, x*(out_gen_pois$sigma), col='orange')              # generalized Poisson
legend('topleft',
       col=c('black','blue','red','green','magenta','orange'),
       lty=c(1,1,1,1,1,1),
       legend=c('Poisson','NB1','NB2','Gamma (log)','Gaussian inverse','Generalized Poisson'),
       cex=0.5)



# model fit (using DHARMa package)    ## Poisson model demonstrates overdispersion; other models demonstrate good fit
check_pois <- simulateResiduals(fittedModel=pois, n=1000)
plot(check_pois)
testDispersion(check_pois)

check_nb1 <- simulateResiduals(fittedModel=nb1, n=1000)
plot(check_nb1)
testDispersion(check_nb1)

check_nb2 <- simulateResiduals(fittedModel=nb2, n=1000)
plot(check_nb2)
testDispersion(check_nb2)

check_gamma <- simulateResiduals(fittedModel=gamma_log, n=1000)
plot(check_gamma)
testDispersion(check_gamma)

check_invg <- simulateResiduals(fittedModel=inv_gauss, n=1000)
plot(check_invg)
testDispersion(check_invg)

check_genpois <- simulateResiduals(fittedModel=gen_pois, n=1000)
plot(check_genpois)
testDispersion(check_genpois)


#### AIC between models (little difference between models that allow overdispersion)
AIC(pois)  ## highest value
AIC(nb1)
AIC(nb2)
AIC(gamma_log)
AIC(inv_gauss)
AIC(gen_pois)


### McFadden between models (little difference between models with overdispersion)
```

© 2023 Decker KM et al. *JAMA Network Open.*

```
null <- glm(count ~ offset(log(stdpop)), family='poisson', data=inc)
McFad_1 <- function(mod){
  return(round(1-((logLik(mod)[1]-mod$rank)/logLik(null)[1]),3))
}
McFad_2 <- function(mod){
  return(round(1-((logLik(mod)[1]-(length(mod$fit$par)-1))/logLik(null)[1]),3))
}

McFad_1(pois)  ## lowest value
McFad_2(nb1)
McFad_1(nb2)
McFad_1(gamma_log)
McFad_1(inv_gauss)
McFad_2(gen_pois)



##############################################
###   check for monthly/seasonal effects
##############################################

## NB1 model selected from overdispersed models

nb1 <- glmmTMB(count ~ offset(log(stdpop)) + covid1*time + covid2*time + ns(month, df=6),   ## splines used on
month variable
              data=inc, family=nbinom1)
McFad_2(nb1)                                                    ## R^2 decreases and then increases (df=5 and 6
include warnings)

check_nb1 <- simulateResiduals(fittedModel=nb1, n=1000)
plot(check_nb1)
testDispersion(check_nb1)                                       ## df=5 and 6 demonstrate poorer fit than without
monthly/seasonal effect included


#####################################################
###        Final ITS model
#####################################################

### NB1 model selected without monthly/seasonal effect included

model <- glmmTMB(count ~ offset(log(stdpop)) + covid1*time + covid2*time, data=inc, family=nbinom1)

summary(model)
confint(model) # 95% CI
Anova(model, type='III')

##
## try including a typical covid*time interaction instead of dummy covid variables,
## modifying the rest of the code, and produce the ITS plot to see the impact





##############################################
###     Calculate ratios and 95% CI
##############################################

inc_ratio <- inc

model_ratio <- glmmTMB(count ~ offset(log(stdpop)) + covid1*time + covid2*time,
```

© 2023 Decker KM et al. *JAMA Network Open.*

```
                  data=inc_ratio, family=nbinom1)

summary(model_ratio)
colnames(getME(model_ratio, name='X'))                    ## determines values to put in matrix (intercept,
intervention, time)
#names(coef(model_ratio))                                 ## alternative when using non-glmmTMB model

# add covid:time column for interactions
inc_ratio$covid1_time <- inc_ratio$covid1 * inc_ratio$time
inc_ratio$covid2_time <- inc_ratio$covid2 * inc_ratio$time


# loop to extract ratios and 95% CI when including an interaction term
first_time <- 64    # value for the first time value during COVID
last_time <- 80     # value for the last time value during COVID

mean <- NULL   # NULL vectors to capture values in loop
se <- NULL
time <- NULL

for(i in first_time:last_time) {

  inc_ratio_int <- subset(inc_ratio, time==i)       # i refers to number in loop (from "first_time" to "last_time")

  K1 <- with(inc_ratio_int,(matrix(c(0, covid1, time, covid2, covid1_time, covid2_time), 1)))
  K2 <- with(inc_ratio_int,(matrix(c(0, 0, time, 0, 0, 0), 1)))

  K <- K1 - K2

  t <- glht(model_ratio, linfct = K)                  # calls model object and comparison matrices
  out <- summary(t, test = adjusted('none'))

  mean1 <- out$test$coefficients
  se1 <- out$test$sigma

  time <- c(time,i)                                   # fill NULL vectors with extracted values
  mean <- c(mean,mean1)
  se <- c(se,se1)

}

# ratios and 95% CI
ratio_output <- as.data.frame(cbind(mean,se,time))
ratio_output$ratio <- exp(ratio_output$mean)
ratio_output$low_CI <- exp(ratio_output$mean - (1.96*ratio_output$se))
ratio_output$high_CI <- exp(ratio_output$mean + (1.96*ratio_output$se))

# fitted values
pred <- predict(model, type='response', newdata=inc)
pred[63] <- NA   ## set March 2020 predictions to NA

# counterfactual predictions
pred_counter <- predict(model, type='response', newdata=inc_counter)
pred_counter[63] <- NA   ## set March 2020 predictions to NA

inc3 <- cbind(inc, pred, pred_counter)

# calculate 95% CI for predicted values in COVID period (pred_counter*ratio CI limits)
inc3_ratio <- merge(inc3, ratio_output, by='time', all.x=TRUE)
inc3_ratio$pred_lower <- with(inc3_ratio, ifelse(covid==1, pred_counter * low_CI, NA))
inc3_ratio$pred_upper <- with(inc3_ratio, ifelse(covid==1, pred_counter * high_CI, NA))
```

© 2023 Decker KM et al. *JAMA Network Open.*

```
########################################
###         ITS Plot
########################################

box <- data.frame(x1=63,x2=84,y1=0,y2=60)
ggplot(data=inc3_ratio) +
  geom_rect(data=box, aes(xmin=x1,xmax=x2,ymin=y1,ymax=y2,fill='grey'), alpha=0.3) +
  geom_point(aes(x=time, y=stdrate, shape=1)) +
  geom_line(aes(x=time, y=pred/stdpop*100000, colour='red')) +
  geom_ribbon(data=subset(inc3_ratio, covid==1), aes(x=time, ymin=pred_lower/stdpop*100000,
ymax=pred_upper/stdpop*100000, fill='red'), alpha=0.2) +
  geom_line(data=subset(inc3_ratio, covid==1), aes(x=time, y=pred_counter/stdpop*100000, linetype='solid')) +
  scale_y_continuous(expand=c(0,0), limits=c(0,60), breaks=seq(0,60,10)) +
  scale_x_continuous(expand=c(0,0), limits=c(0,84),
                     breaks=c(seq(0,84,6)),
                     labels=c('','2015','','2016','','2017','','2018','','2019','','2020','','2021','')) +
  labs(x='Years', y='Age-standardized Rate (per 100,000)') +
  theme_bw() +
  theme(panel.grid.major=element_blank(),
        panel.grid.minor=element_blank(),
        panel.border=element_blank(),
        axis.line=element_line(colour='black'),
        axis.ticks.x=element_line(colour=c('black',rep(c(NA,'black'),7))),
        plot.title=element_text(hjust=0.5),
        legend.position='bottom',
        legend.title=element_blank()) +
  guides(colour=guide_legend(order=3),
         fill=guide_legend(order=4),
         linetype=guide_legend(order=2),
         shape=guide_legend(order=1)) +
  scale_colour_identity(guide='legend', label='Fitted') +
  scale_fill_identity(guide='legend', labels=c('95% CI','COVID-19 Period'), breaks=c('red','grey')) +
  scale_linetype_identity(guide='legend', label='Predicted') +
  scale_shape_identity(guide='legend', label='Observed')



##########################################################################
#####       Cumulative deficits through parametric bootstrapping
##########################################################################

inc_deficit <- subset(inc3_ratio, covid==1, select=c('year','month','pred','pred_counter'))

inc_deficit$cum_pred <- cumsum(inc_deficit$pred)  ## cumulative fitted
inc_deficit$cum_pred_counter <- cumsum(inc_deficit$pred_counter)  ## cumulative predicted
inc_deficit$cum_diff <- with(inc_deficit, cum_pred - cum_pred_counter)  ## cumulative difference
inc_deficit$cum_per_diff <- with(inc_deficit, cum_diff/cum_pred_counter*100)  ## cumulative % difference


M <- 1000  ## number of simulations
boot_cum_def <- NULL        ## null vector to capture cumulative deficit %

# Simulation loop
for(i in 1:M) {

  boot_data <- simulate(model, nsim=1, seed=i)                              ## seed number is updated with simulation
number

  boot_data1 <- boot_data[1:62,]                                            ## data up to Feb 2020
  boot_data2 <- c(NA)                                                       ## data for Mar 2020
```

```
  colnames(boot_data2) <- colnames(boot_data1)                          ## data for Apr 2020+
  boot_data3 <- boot_data[63:79,]

  boot_outcome <- as.data.frame(c(boot_data1,boot_data2,boot_data3))  ## simulated data with proper NAs
  colnames(boot_outcome) <- c("boot_outcome")
  boot_dataset <- cbind(boot_outcome,inc)                               ## dataset with simulated outcome and
original predictors

  ## analyze simulated outcome with same GLM model as final model and parameters
  model_boot <- glmmTMB(boot_outcome ~ offset(log(stdpop)) + covid1*time + covid2*time,
                  data=boot_dataset, family=nbinom1)

  ## produce predictions
  pred_sim <- predict(model_boot, type='response', newdata=boot_dataset)
  pred_sim[63] <- NA   ## set March 2020 predictions to NA

  # counterfactual predictions
  pred_counter_sim <- predict(model_boot, type='response', newdata=inc_counter)
  pred_counter_sim[63] <- NA   ## set March 2020 predictions to NA

  ## attach predictions and counterfactual to original data
  boot_pred <- cbind(inc, pred_sim, pred_counter_sim)

  # cumulative deficit
  inc_deficit_sim <- subset(boot_pred, covid==1, select=c('year','month','pred_sim','pred_counter_sim'))

  inc_deficit_sim$cum_pred <- cumsum(inc_deficit_sim$pred_sim)  ## cumulative predicted
  inc_deficit_sim$cum_pred_counter <- cumsum(inc_deficit_sim$pred_counter_sim)  ## cumulative expected
  inc_deficit_sim$cum_diff <- with(inc_deficit_sim, cum_pred - cum_pred_counter)  ## cumulative difference
  inc_deficit_sim$cum_per_diff <- with(inc_deficit_sim, cum_diff/cum_pred_counter*100)  ## cumulative % difference

  boot_cum_def <- c(boot_cum_def,inc_deficit_sim$cum_per_diff)      ## save percentage
}

## modifiy saved output to create dataset
out <- matrix(boot_cum_def, nrow=17, ncol=M, byrow=F)   ## 17 rows for the number of months in the pandemic
calculated, M columns for simulated output


## loop function to calculate percentile values across rows
out_q <- NULL

for(i in 1:17) {  ## max number (17) is number of rows in "out" matrix
  outq <- quantile(out[i,], probs=c(0.025,0.975))
  out_q <- c(out_q,outq)
}

## transform out_q into dataframe
deficit_CI <- as.data.frame(matrix(out_q, nrow=17,ncol=2, byrow=T))
colnames(deficit_CI) <- c('cum_per_diff_lower','cum_per_diff_upper')

inc_deficit2 <- cbind(inc_deficit,deficit_CI)
inc_deficit2
```

© 2023 Decker KM et al. *JAMA Network Open.*
