# Supplementary material for: New Cancer Diagnoses Before and During the COVID-19 Pandemic
Source: JAMA Netw Open. 2023 Sep 5;6(9):e2332363. doi: 10.1001/jamanetworkopen.2023.32363 (PMC10481240; doi:10.1001/jamanetworkopen.2023.32363)
Supplement: Supplement 2. — Data Sharing Statement [file jamanetwopen-e2332363-s002.pdf]

Data Sharing Statement

Decker. New Cancer Diagnoses Before and During the COVID-19 Pandemic. *JAMA Netw Open*. Published September 05, 2023. doi:10.1001/jamanetworkopen.2023.32363

Data
Data available: No

Additional Information
Explanation for why data not available: The data that support the findings of this study are not publicly available to ensure and maintain the privacy and confidentiality of individuals' health information. Requests for data may be made to the appropriate data stewards (CancerCare Manitoba's Research and Resource Impact Committee).
